# Enhancing human pluripotent stem cell differentiation to cardiomyocytes through cardiac progenitor reseeding and cryopreservation

**DOI:** 10.1016/j.isci.2025.112452

**Published:** 2025-04-16

**Authors:** Austin K. Feeney, Aaron D. Simmons, Claire J. Peplinski, Xiaotian Zhang, Sean P. Palecek

**Affiliations:** 1 Department of Biomedical Engineering, University of Wisconsin-Madison, Madison, WI 53706, USA; 2 Medical Scientist Training Program, University of Wisconsin School of Medicine and Public Health, Madison, WI 53726, USA; 3 Department of Chemical and Biological Engineering, University of Wisconsin-Madison, Madison, WI 53706, USA

**Keywords:** Biological sciences, Technical aspects of cell biology, Biological sciences research methodologies

## Abstract

Human pluripotent stem cell-derived cardiomyocytes (hPSC-CMs) have the potential to transform the understanding of heart development and heart failure treatment. However, hPSC-CM differentiation efficiency is plagued by batch-to-batch and line-to-line variability. Here, we describe a method to improve CM purity by 10–20% (absolute) without negatively affecting contractility, sarcomere structure, multinucleation, junctional Cx43, or CM number by detaching and reseeding progenitors between the *EOMES*+ mesoderm and *ISL1*+/*NKX2-5*+ cardiac progenitor stages. Moreover, we demonstrate that *EOMES*+ mesoderm and *ISL1*+/*NKX2-5*+ cardiac progenitors are cryopreservable with similar improvements in CM purity after resuming differentiation, facilitating storage of large batches of hPSC-CM progenitors for on-demand CM production. Reseeding during differentiation also enables transition to defined extracellular matrices, including fibronectin, vitronectin, and laminin-111, which all supported hPSC-derived *EOMES*+ mesoderm and *ISL1*+/*NKX2-5*+ cardiac progenitor differentiation to CMs. In summary, we present a method to increase hPSC-CM differentiation purity and demonstrate that specific CM progenitors are amenable to cryopreservation.

## Highlights

• Reseeding *EOMES*+ mesoderm and *ISL1*+/*NKX2-5*+ CPCs improved hPSC-CM purity by 10–20%
• Reseeding CM progenitors had minimal impact on contractility and sarcomere length
• *EOMES*+ mesoderm and *ISL1*+/*NKX2-5*+ CPCs differentiated to CMs after cryopreservation
• Reseeding cryopreserved *EOMES*+ mesoderm and *ISL1*+/*NKX2-5*+ CPCs improved CM purity

## Graphical abstract

**Figure:**
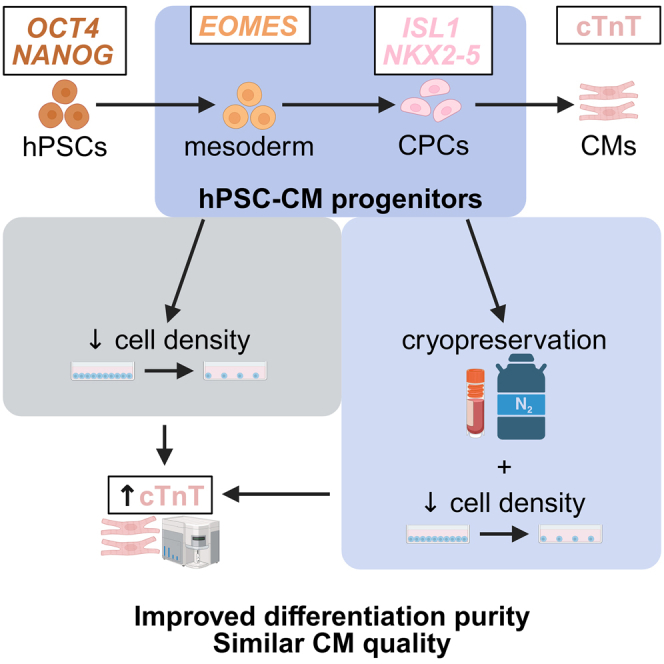

## Introduction

Myocardial infarction causes pathological ventricular remodeling characterized by cardiomyocyte death and non-contractile, fibrotic scar formation. Human cardiomyocytes do not regenerate appreciably *in vivo*, and no current treatments promote remuscularization following cardiac injury.^1^ As a result, human pluripotent stem cell-derived cardiomyocytes (hPSC-CMs) provide an unparalleled opportunity to replace lost cardiomyocytes via cellular therapy in addition to their promise for drug discovery and disease modeling applications.^2^^,^^3^^,^^4^^,^^5^^,^^6^^,^^7^ Directed differentiation strategies to generate hPSC-CMs generally strive to result in 75–99% pure CMs; however, batch-to-batch variability results in high batch failure rates, increasing the cost and time to generate batches that meet quality standards.^8^^,^^9^ Unfortunately, we have limited ability to predict CM purity early in the differentiation process, and there are few consensus methods to increase differentiation purity across multiple cell lines and differentiation protocols.

Prior tissue engineering and pre-clinical transplantation studies have demonstrated that high purity (≥70% cTnT+) CM formulations improve contractility *in vitro* and *in vivo* following transplantation.^2^^,^^3^^,^^10^ However, numerous studies and laboratories have reported differentiation outcomes anywhere from 1 to 97% cTnT+ CMs, with typical differentiations achieving between 30 and 70% cTnT+ CMs or an average CM purity of around 50–60%.^11^^,^^12^^,^^13^^,^^14^^,^^15^^,^^16^ As such, developing protocol advancements that improve average CM purity by 10–20% (absolute) would have profound impacts on the number of successful differentiations that reach a high purity threshold of ≥70% cTnT+ CMs for downstream applications. While metabolic and immunologic methods for CM purification enable the preparation of high-purity CM formulations, neither of these approaches fundamentally increases the number of CMs per hPSC and instead results in the incomplete recovery of CMs from hPSC-CM differentiations, reducing the CM number.^17^^,^^18^^,^^19^ The requirement to reproducibly manufacture high purity CMs for clinical translation and research applications demonstrates the importance of making adaptations to hPSC-CM differentiation protocols that improve purity across multiple cell lines, reduce the need for terminal CM purification, and allow for the prediction of terminal CM purity.

Given the variability of hPSC-CM differentiation and the requirement for substantial cell doses in cell therapy applications, several groups have explored the cryopreservation of hPSC-CMs or cardiac progenitor stage cells (CPCs).^20^^,^^21^^,^^22^^,^^23^ Cryopreservation of hPSC-CMs typically results in 60–90% cell recovery in a variety of freezing media.^2^^,^^22^^,^^23^^,^^24^^,^^25^ Additionally, cryopreserved CMs have demonstrated similar purity, molecular characteristics, contractile properties, electrophysiology, and transplantation success as non-cryopreserved controls.^2^^,^^23^^,^^26^^,^^27^ Fewer studies have investigated the cryopreservation of hPSC-CM progenitors.^20^^,^^21^^,^^28^ Yet, those studies have demonstrated 70–90% cell recovery after cryopreservation and a retained ability of CPCs to successfully differentiate into cardiomyocytes. Furthermore, FUJIFILM Cellular Dynamics International currently sells cryopreserved KDR+/PDGFRα+ CPCs that have the ability to differentiate into spontaneously beating cardiomyocytes upon the addition of Wnt and TGFβ inhibition.^29^^,^^30^ One previous study also demonstrated the ability to cryopreserve *MESP1*+ mesoderm stage cells during hPSC-CM differentiation; however, they achieved inefficient differentiation to cardiomyocytes.^21^ However, no prior studies have systematically compared both recovery and CM differentiation capacity of progenitors at different stages of hPSC-CM differentiation.

In this study, we developed a simple hPSC-CM differentiation protocol adaptation to increase CM purity by 10–20% (absolute) while maintaining CM number across multiple cell lines and differentiation protocols by detaching and reseeding CM progenitors at a lower density. This method did not substantially impact terminal sarcomere structure, multinucleation, the ability to express junctional Cx43 in CMs, or contractility parameters, including beat rate and contraction or relaxation duration. Additionally, myosin heavy chain isoform expression (MYH7/MYH6) either remained unchanged after reseeding or shifted toward a more mature cardiomyocyte phenotype characterized by an increase in the percentage of single-positive MYH7 and a subsequent decrease in the percentage of double-positive MYH7/MYH6 expressing cardiomyocytes. Moreover, we specifically show that *EOMES*+ mesoderm progenitors and *ISL1*+/*NKX2-5*+ CPCs exhibit high recovery following cryopreservation and that reseeding these cryopreserved progenitors also enhances CM purity compared to non-cryopreserved controls. Furthermore, we demonstrate that this reseeding approach enables the precise temporal introduction of developmentally relevant defined extracellular matrix (ECM) proteins.^31^^,^^32^^,^^33^^,^^34^ By cryopreserving *EOMES*+ mesoderm progenitors or *ISL1*+/*NKX2-5*+ CPCs, one can generate large batches of CM-fated progenitors. This facilitates quality control of CM progenitors and on-demand CM production from these cryopreserved intermediates.

## Results

### Reseeding at the cardiac progenitor stage of human pluripotent stem cell-derived cardiomyocyte differentiation improves cardiomyocyte purity

We previously generated a small molecule-based hPSC-CM differentiation protocol, known as the GiWi protocol, that relies solely on the modulation of the Wnt signaling pathway.^32^^,^^35^ This protocol first guides hPSCs to the mesoderm fate through Wnt activation using CHIR99021, a glycogen synthase kinase 3 inhibitor. Subsequently, cardiac mesoderm is formed through Wnt inhibition using IWP2, a porcupine inhibitor. While high purity hPSC-CM differentiation (>70% cTnT+) is attainable using the GiWi protocol, terminal CM purity is highly dependent on using an optimal initial cell seeding density and CHIR99021 concentration.^8^^,^^36^

Given the variability in hPSC-CM differentiation purity and the importance of cell density in early CM differentiation, we investigated whether detaching and reseeding CM progenitors at a lower density could improve CM purity during hPSC-CM differentiation. To assess the effects of reseeding CPCs at a lower density during hPSC-CM differentiation, we plated cryopreserved WTC11 CPCs (D5) at 1:1, 1:2.5, 1:5, and 1:10 ratios by surface area for 3 independent differentiations (initial differentiation surface area to reseeded surface area). Then, we assessed the resulting terminal CM purity (% of cTnT+ cells), number of CMs Relative to CTRL, absolute percent cTnT+ difference from CTRL, cell confluency, and contractile characteristics using MUSCLEMOTION (beats per minute, relaxation duration, and contraction duration) on day 16 (Figure S1).^37^

**Figure 1:**
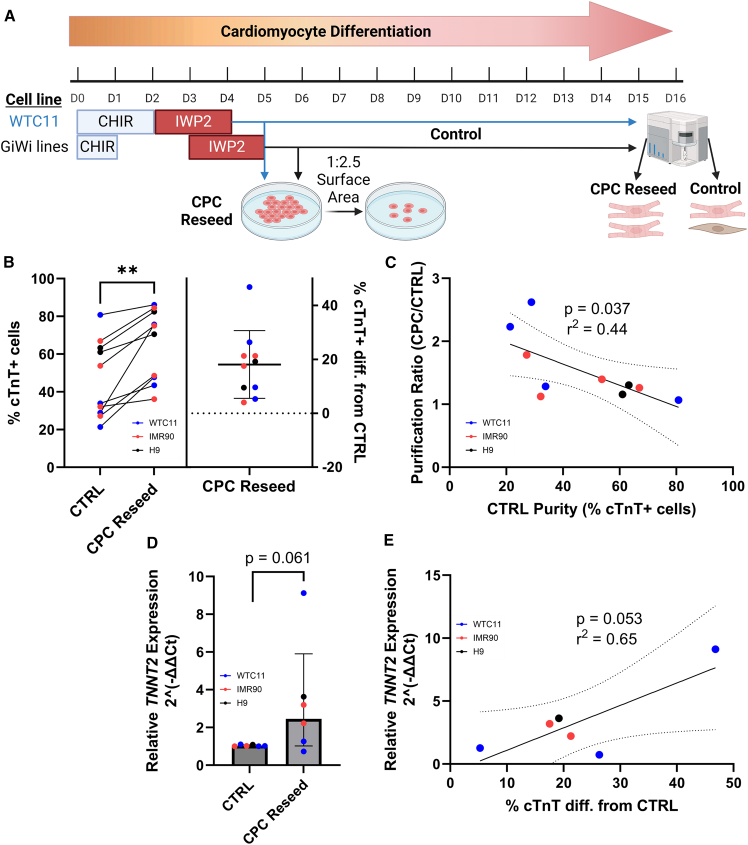
Reseeding at the cardiac progenitor stage of hPSC-CM differentiation improves cardiomyocyte purity (A) Overview of the cardiomyocyte differentiation across cell lines (WTC11 in blue, GiWi lines in black: IMR90-4, H9, and 19-9-11) and the cardiac progenitor cell (CPC) reseeding protocol (CHIR = CHIR99021). (B) Flow cytometry analysis of cTnT expression in D16 hPSC-CMs for control (CTRL) differentiation samples compared to CPC reseed samples in 3 cell lines indicated in different colors. Points (left portion) represent the mean values of 3–4 technical replicates for 10 independent differentiations (4 WTC11, 4 IMR90-4, 2 H9). Points (right portion) represent the absolute difference in the percentage of cTnT+ cells in the CPC reseed condition and the CTRL condition for each differentiation replicate across cell lines represented by distinct colors. *p*-value from paired t-test. (C) Linear regression of CTRL purity versus purification ratio, which is the CPC Reseed purity divided by the CTRL purity. Points represent the mean values of 3–4 technical replicates for 10 independent differentiations (4 WTC11, 4 IMR90-4, 2 H9). The solid line represents the best-fit line, and the dashed lines represent the 95% confidence intervals. *p*-value and r^2^ value from simple linear regression. (D) RT-qPCR of relative *TNNT2* expression (2^[-ΔΔCt]^) for CTRL differentiation samples compared to CPC reseeded samples one to two days after CPC reseed recovery (D6 for WTC11, D7 for H9, D7-8 for IMR90-4). Points represent the mean values of 2–3 differentiation well replicates for 6 independent differentiation represented by distinct colors (3 WTC11, 2 IMR90-4, 1 H9). *p*-value from paired t-test. (E) Linear regression of % cTnT diff. from CTRL from flow cytometry versus relative *TNNT2* expression (2^[-ΔΔCt]^) from RT-qPCR. Points represent the mean values of 2–4 technical replicates for 6 independent differentiations represented by distinct colors (3 WTC11, 2 IMR90-4, 1 H9). The solid line represents the best-fit line, and the dashed lines represent the 95% confidence intervals. *p*-value and r^2^ value from simple linear regression. ∗ for *p* < 0.05, ∗∗ for *p* < 0.01, ∗∗∗ for *p* < 0.001, ∗∗∗∗ for *p* < 0.0001, and ns for *p* > 0.05. All data are represented as mean ± SD.

To identify single cardiomyocytes, we used flow cytometry to gate cells on FSC-A vs. SSC-A and then gated single cells on both FSC-A vs. FSC-H and SSC-A vs. SSC-H (Figure S1A). Then, we identified cTnT+ cardiomyocytes by comparing hPSC-CM differentiation samples to an undifferentiated hPSC negative control population and gated cTnT+ cardiomyocytes on FSC-A vs. Alexa Fluor 488 (Figure S1A). cTnT purity significantly increased in comparison to the CTRL differentiation for CPC reseeding at 1:1, 1:2.5, and 1:5 ratios (Figure S1B). However, cTnT purity significantly decreased with reseeding at a 1:10 ratio, which indicated that there is an upper limit to the beneficial effects of lowering cell density. The number of cardiomyocytes relative to the CTRL differentiation was significantly lower when reseeding at 1:1, 1:5, and 1:10 ratios but remained unchanged when reseeding at a 1:2.5 ratio (Figure S1C). The mean cTnT+ purity increase (absolute) from CTRL was roughly 12% at the 1:2.5 ratio and 15% at the 1:5 ratio (Figure S1D). Additionally, cell confluency and morphology were assessed on D16 using phase contrast imaging (Figure S1E). Cell confluency reached 100% for reseeding at all ratios except 1:10, where confluency only reached 60%. In reseeded conditions, there appeared to be fewer cell layers even though confluency reached 100%, and morphology appeared slightly different but more homogeneous throughout the well in comparison to the CTRL differentiation. Lastly, to evaluate if reseeding CPCs influenced cardiomyocyte contractile properties, we acquired videos of spontaneously beating cultures prior to collection for flow cytometry. These videos were automatically analyzed using MUSCLEMOTION to assess spontaneous beat rate, contraction duration, and relaxation duration (Figures S1F–S1H). Although spontaneous beating was observed in all conditions, MUSCLEMOTION only accurately quantified beating for all 3 differentiations for the CTRL, 1:1, and 1:2.5 conditions. Beat rate was significantly decreased in the 1:1 reseed condition but remained unchanged in the 1:2.5 reseed condition in comparison to the CTRL (Figure S1F). Relaxation duration and contraction duration were unchanged after reseeding at both the 1:1 and 1:2.5 ratios in comparison to the CTRL (Figures S1G and S1H). Collectively, these data demonstrated that reseeding at the 1:2.5 ratio resulted in the best combination of cTnT purity improvement, cardiomyocyte number, cell recovery, and contractile properties. As such, the 1:2.5 reseeding ratio was selected for additional CPC reseeding experiments spanning multiple cell lines (Figure 1).

The CPC stage was defined one day following the removal of IWP2 treatment, which is at D5 for WTC11 iPSCs and at D6 for all GiWi cell lines (IMR90-4 and 19-9-11 iPSCs, H9 hESCs) used in this study (Figure 1A). The differential timing for Wnt signaling modulation in WTC11 iPSC CM differentiation is based on the Allen Cell Institute WTC11 optimized protocol for CM differentiation (SOP: Cardiomyocyte differentiation methods_v1.2.pdf), which has been reported by others and in our prior work.^38^^,^^39^^,^^40^^,^^41^ For 10 independent differentiations in 3 cell lines, we found that reseeding CPCs resulted in a significant increase in the percentage of cTnT+ cardiomyocytes for all differentiations with a mean 20% absolute increase in CM purity (Figures 1B, S2A, and S2B). Notably, the ability to increase CM purity by reseeding CPCs increased with decreasing CTRL purity up to a 2.5-fold increase in CM purity (Figure 1C). The effects of media composition on CM purity during CPC reseed were also examined, and it was determined that there was no clear benefit of adding insulin or ROCK inhibitor Y-27632 on CM purity (Figure S2C). Moreover, changing media at the CPC stage without reseeding did not result in an increase in purity, and actually decreased purity when insulin was introduced in line with previous reports (Figure S2C).^42^^,^^43^

To investigate the effect of CPC reseeding on the expression of canonical CM and CPC markers, we performed an RT-qPCR experiment to quantify changes in *TNNT2* (CM), *ISL1*, *NKX2-5*, and *MEF2C* expression. For this experiment, we evaluated the expression of CM and CPC markers 1–2 days after reseeding in comparison to day-matched CTRL differentiation samples for 3 WTC11 differentiations and 3 GiWi line differentiations (1 H9, 2 IMR90-4). CPC reseeding led to an immediate increase in TNNT2 expression in comparison to day-matched CTRL differentiation samples (*p* = 0.061, Figure 1D). Moreover, the relative *TNNT2* gene expression immediately after reseeding linearly tracked with the D16 increase in cTnT purity by flow cytometry (*p* = 0.053 and r^2^ = 0.65, Figure 1E). For CPC markers, *ISL1* expression was unchanged after reseeding; however, *NKX2-5* and *MEF2C* expression were significantly reduced in comparison to day-matched CTRL differentiation samples (Figures S2D–S2F). Together, these data suggest that CPC reseeding corresponded with a shift from CPC marker expression toward *TNNT2* expression.

All cell lines recovered after CPC reseed and established roughly 80–100% confluency by D16. Representative images tracking CPC recovery from a low efficiency and high efficiency CTRL differentiation are shown for IMR90-4 and WTC11 iPSC lines, respectively (Figures S3A and S3B). Morphology between CTRL and CPC reseed D16 cultures was slightly different, with reseeded cultures having more branching and dense cell clusters than CTRL differentiation samples, which had a more sheet-like appearance (Figures S3A and S3B). However, morphology varied between differentiation batches for D16 CTRL and CPC reseed samples.

### Reseeding early progenitors of human pluripotent stem cell-derived cardiomyocyte differentiation improves cardiomyocyte purity

To better define the early stages of hPSC-CM differentiation prior to extending the reseeding approach to earlier time points, we performed a temporal RT-qPCR experiment investigating the expression of markers corresponding to the following stages of differentiation: undifferentiated (*POU5F1*, *NANOG*), mesoderm (*TBXT*, *EOMES*), cardiac progenitor (*KDR*, *PDGFRA*, *ISL1*, *NKX2-5*, *TBX5*, *MEF2C*, *GATA4*) and cardiomyocyte (*TNNT2*). We profiled the expression of these molecular markers from D0-D6 for 3 independent WTC11 differentiations and from D0-D7 for 3 independent GiWi line differentiations (2 H9, 1 IMR90-4) for CTRL differentiations with D16 cTnT purity greater than 60%. The expression of undifferentiated markers *POU5F1* and *NANOG* was highest on D0 (prior to CHIR99021 addition) and significantly decreased across cell lines within the first few days of differentiation (Figures S4A–S4D). While the dynamics of *TBXT* expression varied between cell lines, mesodermal marker *EOMES* exhibited the highest expression on the day of IWP2 addition across cell lines (D2 WTC11 and D3 GiWi lines). Thus, progenitor cells at this stage are hereafter referred to as *EOMES*+ mesoderm. One day after IWP2 addition (D3 WTC11 and D4 GiWi lines), cells expressed a combination of the mesodermal marker *EOMES* and cardiac progenitor markers *PDGFRA*, *ISL1*, and *GATA4* cell lines. Because of this, progenitor cells at this stage are hereafter referred to as cardiac mesoderm. Cardiac progenitor markers *PDGFRA* and *KDR* exhibited the highest co-expression on the day of IWP2 removal across cell lines (D4 WTC11 and D5 GiWi lines), and progenitor cells at this stage are identified as *KDR*+/*PDGFRA*+ CPCs. Of note, *KDR*+/*PDGFRA*+ CPCs were also highly positive for cardiac progenitor markers *ISL1*, *TBX5* and *GATA4*. On D5 in WTC11 and D6 in GiWi lines (reseed day used in Figure 1, one day after IWP2 removal), all cardiac progenitor markers were increased in expression in comparison to D0 samples except for *KDR*. This was exemplified by high *ISL1* expression and early *NKX2-5* and *MEF2C* expression. Because of this, progenitor cells at this stage are hereafter referred to as *ISL1*+/*NKX2-5*+ CPCs. Of note, this day also corresponded with the earliest day of substantial *TNNT2* expression even though spontaneous beating was not visible in cultures at this time.

After observing increased CM purity following reseeding D5/6 *ISL1*+/*NKX2-5*+ CPCs, we hypothesized that reseeding earlier progenitors during hPSC-CM differentiation may also be possible and may further enhance terminal CM purity. Moreover, we hypothesized that reseeding hPSC-CM progenitors may also improve the number of CMs generated, as proliferative markers are higher during early phases of differentiation.^41^^,^^44^ Furthermore, a previous publication reported that the removal of cell-cell contacts in combination with Wnt signaling activation can increase the proliferation of Day 12 hPSC-CMs by preventing contact-mediated cell cycle inhibition through the activation of Wnt and Akt signaling.^45^^,^^46^ To test the effects of reseeding early CM progenitors at a lower density, we compared CM purity and the number of CMs relative to CTRL after reseeding *EOMES*+ mesoderm, cardiac mesoderm, *KDR*+/*PDGFRA*+ CPCs, and *ISL1*+/*NKX2-5*+ CPCs at a 1:2.5 surface area ratio for WTC11 and GiWi cell lines. Reseeding hPSC-CM progenitors after the *ISL1*+/*NKX2-5*+ CPC stage at the 1:2.5 surface area ratio resulted in poor recovery, potentially due to cell death, poor attachment, or lack of proliferation (data not shown).

**Figure 2:**
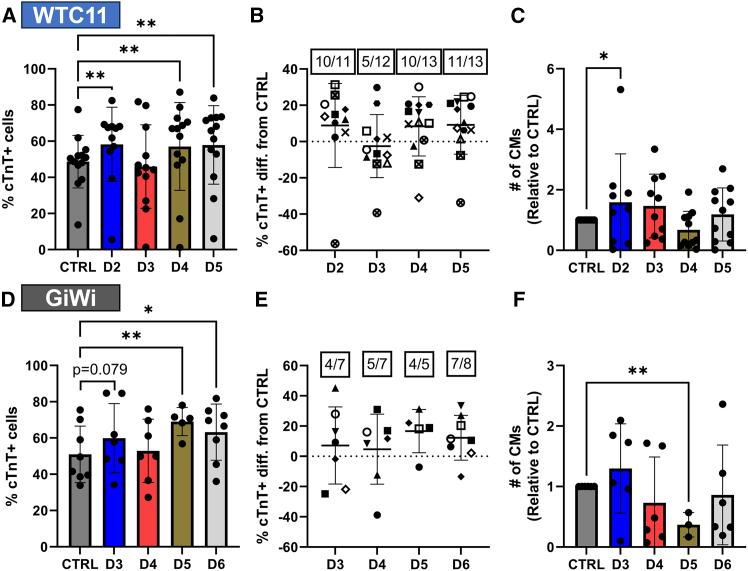
Reseeding early progenitors of hPSC-CM differentiation improves cardiomyocyte purity (A) Flow cytometry analysis of cTnT expression in hPSC-CMs for control (CTRL) differentiation samples compared to reseed samples at the indicated days in the WTC11 iPSC line (A-C). Points represent the mean values of 3–6 technical replicates for 13 independent differentiations. (B) Absolute difference in the percentage of cTnT+ cells for the indicated day compared to the CTRL of the same differentiation. Unique symbols represent independent differentiations. Fractions at the top of the graph represent the number of differentiations out of the total number of differentiations that increased in percentage of cTnT+ cells (difference from CTRL >0). (C) The number of CMs relative to CTRL differentiation samples. Unique symbols represent the mean values of 3–6 technical replicates for 11 independent differentiations. (D) Flow cytometry analysis of cTnT expression in hPSC-CMs for control (CTRL) differentiation samples compared to reseed samples at the indicated days in IMR90-4, H9, and 19-9-11 hPSC lines (D–E). Points represent the mean values of 3-4 technical replicates for 8 independent differentiations (6 IMR90-4, 1 H9, 1 19-9-11). (E) Absolute difference in the percentage of cTnT+ cells for the indicated day compared to the CTRL of the same differentiation. Unique symbols represent independent differentiations. Fractions at the top of the graph represent the number of differentiations out of the total number of differentiations that increased in percentage of cTnT+ cells (difference from CTRL > 0). (F) The number of CMs relative to CTRL differentiation samples. Unique symbols represent the mean values of 3–4 technical replicates for 6 independent differentiations. All *p*-values from two-factor linear mixed-effects model with repeated measures testing for main column effects with a Dunnett’s post-hoc test. ∗ for *p* < 0.05, ∗∗ for *p* < 0.01, ∗∗∗ for *p* < 0.001, ∗∗∗∗ for *p* < 0.0001, and ns for *p* > 0.05. All data are represented as mean ± SD.

We observed significant increases in CM purity when reseeding hPSC-CM progenitors in the WTC11 iPSC line at D2 (*EOMES*+ mesoderm), D4 (*KDR*+/*PDGFRA+* CPCs), and D5 (*ISL1*+/*NKX2-5*+ CPCs) with a mean increase of approximately 10% cTnT+ cells for all 3 conditions (Figures 2A and 2B). Similarly, for GiWi lines, we observed significant increases in CM purity when reseeding hPSC-CM progenitors at D5 (*KDR*+/*PDGFRA*+ CPCs) and D6 (*ISL1*+/*NKX2-5+* CPCs) but no statistical significance for the increase in CM purity at D3 (*EOMES*+ mesoderm, Figure 2D). Taken together, reseeding resulted in a mean absolute increase in CM purity close to 10% for 3 out of 4 days assessed (Figure 2E). Reseeding at the cardiac mesoderm stage, one day after the initiation of IWP2 treatment (D3 for WTC11 or D4 for GiWi cell lines) did not result in an increase in CM purity (Figures 2A and 2D). Notably, these effects were not simply due to the introduction of additional media changes outside of the typical protocols (i.e., changing media on D3 or D5 for WTC11 and D4 or D6 for GiWi lines), as media change controls (wells receiving a medium change on these days without reseeding) did not exhibit similar enhancements in CM purity (Figures S5A and S5D).

Because hPSC-CM purity can be highly variable, a potential benefit of reseeding is the ability to detach cells from multiple plates or wells and pool them prior to reseeding to evenly redistribute cells. To investigate whether this is beneficial in terms of improving cardiomyocyte purity and differentiation reproducibility, we compared the within differentiation standard deviation for the percentage of cTnT+ CMs for differentiations where cells from different wells of a plate were either unpooled (Figures S5B and S5E) or pooled (Figures S5C and S5F) prior to reseeding. On the whole, pooling wells prior to reseeding only significantly reduced within differentiation standard deviation for the percentage of cTnT+ CMs at the *ISL1*+/*NKX2-5*+ CPC stage in GiWi lines (Figure S5F).

To investigate the number of CMs relative to the CTRL differentiation following reseeding, we compared flow cytometry counts in the cTnT+ gate (Figure S1A) between treatments, normalized to the control differentiation samples. For reseeded treatments, CM count in the cTnT+ gate was multiplied by the 1:2.5 split ratio to assess the number of CMs generated per well seeded for differentiation on Day -2. Using this approach, the number of CMs relative to the CTRL significantly increased for D2 (*EOMES*+ mesoderm) reseeding in the WTC11 iPSC line, but not for any other day in either cell line (Figures 2C and 2F). Furthermore, although D5 (*KDR*+/*PDGFRA*+ CPC) reseeding in GiWi lines significantly increased CM purity, it also decreased the number of CMs relative to the CTRL (Figure 2F), which was consistent with lower cell confluency during reseeding recovery prior to day 16 collection (data not shown). Using manual counts on fixed cells prior to flow cytometry, there were no significant differences in the number of CMs relative to the CTRL for any cell line (Figures S6A and S6D). However, the number of CMs relative to the CTRL was highest for *EOMES*+ mesoderm reseeding (D2 for WTC11 and D3 for GiWi lines), consistent with the flow cytometry counts (Figures 2C and 2F).

To understand the differences in the number of CMs relative to CTRL between cell lines, we also manually counted live cells during the reseed process for a subset of the differentiations. These data demonstrate an increase in cell number after Day -2 initial cell seeding prior to the *EOMES*+ mesoderm stage (D2 WTC11 and D3 GiWi, Figures S6B, S6C, and S6E–S6G). While the cell number remained unchanged in the GiWi lines between Days 3–6 in comparison to Day 3 (Figures S6E–S6G), the WTC11 cell number continued to increase between Days 2–5 in comparison to Day 2 (Figures S6B, S6C, S6G). This could be due to the optimal WTC11 Day -2 differentiation seeding density being significantly lower than GiWi lines or potentially that the cells maintained higher proliferative potential at this stage of the differentiation due to slightly altered differentiation protocol timing. Regardless, these data suggest proliferative potential as a mechanism unique to D2 WTC11 (*EOMES*+ mesoderm) reseeding for increased CM number. Additionally, these data suggest that methods to increase CM progenitor proliferation without altering fate commitment could improve CM number relative to the CTRL during progenitor reseeding. However, our data indicate that progenitor reseeding at a 1:2.5 surface area ratio alone largely did not significantly impact CM number across cell lines. To understand cell number during differentiation and during reseed at the 1:2.5 ratio by surface area, manual counts of live cells during the reseed process were divided by 2.5 (Figure S6G). These data demonstrate that the WTC11 reseeding cell density was much lower than GiWi lines at the *EOMES*+ mesoderm stage (D2 WTC11 and D3 GiWi) and at the cardiac mesoderm stage (D3 WTC11 and D4 GiWi) before reaching a similar value at the *KDR*+/*PDGFRA*+ (D4 WTC11 and D5 GiWi) and *ISL1*+/*NKX2-5*+ (D5 WTC11 and D6 GiWi) CPC stages. In summary, reseeding CM progenitors increased CM purity at the *EOMES*+ mesoderm, *KDR*+/*PDGFRA*+ CPC, and *ISL1*+/*NKX2-5*+ CPC stages (3 of the 4 time points assessed) without improving the number of CMs relative to the CTRL across cell lines.

### Reseeding early progenitors of human pluripotent stem cell-derived cardiomyocyte differentiation does not impact contractile function or sarcomere structure

**Figure 3:**
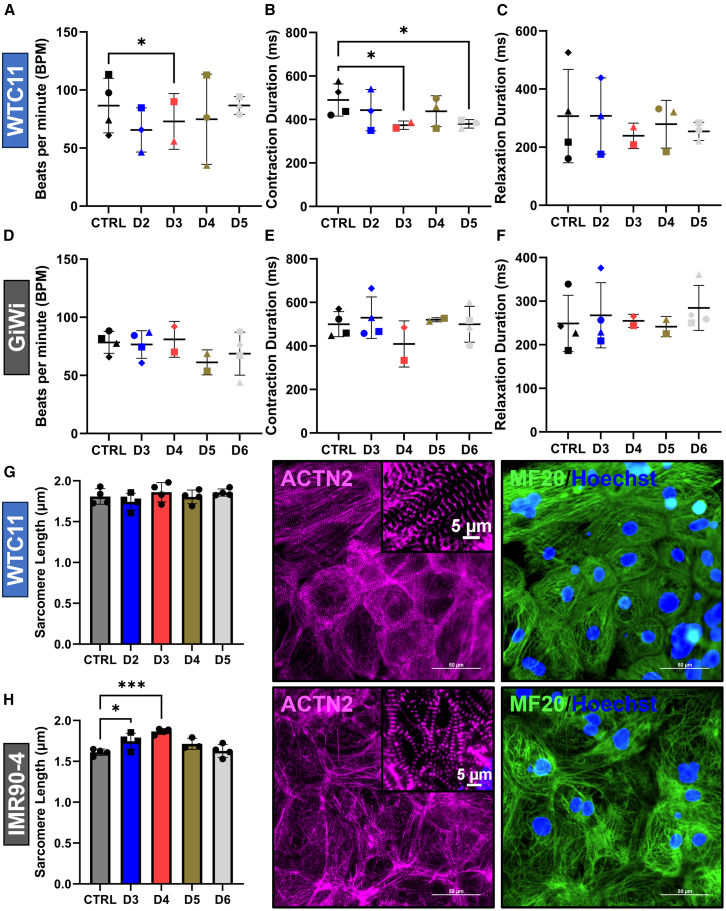
Reseeding early progenitors of hPSC-CM differentiation does not impact contractile function or sarcomere structure (A–C) Contractile parameters (beats per minute - **A**, contraction duration - **B**, and relaxation duration - **C**), quantified using MUSCLEMOTION, in CMs differentiated from the WTC11 iPSC line with reseeding at the indicated time points. Points represent the mean values of 1–5 technical replicates for 4 independent differentiations, which are represented by unique symbols. (D–F) Contractile parameters (beats per minute - **D**, contraction duration - **E**, and relaxation duration - **F**), quantified using MUSCLEMOTION, in CMs differentiated from GiWi cell lines with reseeding at the indicated time points. Points represent the mean values of 1–4 technical replicates for 4 independent differentiations, which are represented by unique symbols (3 IMR90-4, 1 19-9-11). (G) Sarcomere lengths in CMs differentiated from the WTC11 iPSC line, with reseeding at the indicated time points, were quantified at 60x magnification using ImageJ. Points represent the mean values of 4 image replicates for one differentiation. Representative immunofluorescence images with alpha-actinin (magenta), MF20 (green), and Hoechst nuclear (blue) stains. (H) Sarcomere lengths in CMs differentiated from the IMR90-4 iPSC line, with reseeding at the indicated time points, were quantified at 60x magnification using ImageJ. Points represent the mean values of 3–4 image replicates for one differentiation. Representative immunofluorescence images with alpha-actinin (magenta), MF20 (green), and Hoechst nuclear (blue) stains. All images are at 60x magnification with scale bars = 50 μm for the full image and 5 μm for alpha-actinin image insets. *p*-values for A-F from a two-factor linear mixed-effects model with repeated measures testing for a main column effect with a Dunnett’s post-hoc test. *p*-values for G-H from one-way ANOVA with Dunnett’s post-hoc. ∗ for *p* < 0.05, ∗∗ for *p* < 0.01, ∗∗∗ for *p* < 0.001, ∗∗∗∗ for *p* < 0.0001, and ns for *p* > 0.05. All data are represented as mean ± SD.

To evaluate if reseeding hPSC-CM progenitors at different stages influenced cardiomyocyte contractile properties, MUSCLEMOTION was used to assess spontaneous beat rate, contraction duration, and relaxation duration (Figures 3A–3F).^37^ There were no conserved significant differences between reseed conditions and the CTRL differentiation samples between cell lines. For the WTC11 iPSC line, there were minor reductions in beat rate of Day 3 reseeded cells (cardiac mesoderm stage) and contraction duration in Day 3 (cardiac mesoderm stage) and Day 5 (*KDR*+/*PDGFRA*+ CPCs) reseeded cells (Figures 3A and 3B). Relaxation duration was unchanged during progenitor reseeding in the WTC11 iPSC line (Figure 3C). Moreover, there were no significant differences for any of the contractile parameters for the GiWi lines tested (Figures 3D–3F). Beat rate for hPSC-CMs ranged between 40 and 120 beats per minute, consistent with previous reports of hPSC-CMs.^37^^,^^47^^,^^48^

In addition to investigating contractility, we compared the sarcomere structure of reseeded conditions to the control differentiation protocol. To evaluate sarcomere structure, we replated Day 16 cardiomyocytes to obtain single cells and small cell colonies with single-cell thickness and immunostained for the Z-disc protein alpha-actinin 2 following one week of recovery on D23 (Figures 3G and 3H). Cells were co-stained for sarcomeric myosin (MF20) and DNA (Hoechst 33342) to identify cardiomyocytes (Figures 3G and 3H). Sarcomere lengths were manually measured in ImageJ using 60x magnification (see methods). For both cell lines, average sarcomere lengths across conditions ranged between 1.6 and 1.9 μm, consistent with previous reports for hPSC-CMs at this time point with no additional maturation treatment (Figures 3G and 3H).^47^^,^^49^^,^^50^ In the WTC11 iPSC line, there were no significant differences in sarcomere length between the control and reseed conditions (Figure 3G). For the IMR90-4 iPSC line, there was a minor but significant increase in sarcomere length for cells reseeded at the *EOMES*+ mesoderm (D3) and the cardiac mesoderm (D4) stages during the differentiation (Figure 3H). Additionally, we performed the automated quantification of sarcomere length using SotaTool for images at both 60x and 40x magnification (Figures S7A–S7D).^50^ The trends for these results were consistent with our manual sarcomere length quantification, albeit with a slightly higher range for average sarcomere length (1.7–2.0 μm).

To further investigate the CM sarcomere phenotype after reseeding, cells were co-stained for beta myosin heavy chain (MYH7), alpha myosin heavy chain (MYH6), and DNA (Hoechst 33342) and imaged at 20x magnification. We evaluated the percentage of MYH7+/MYH6+ double-positive CMs as well as the percentage of MYH7+ or MYH6+ single positive CMs via immunocytochemistry. The nuclear area was used to define the percentage of CMs in each population. We verified that CM multinucleation was not different across conditions to allow for this analysis (Figures S7E and S7F). The % cTnT cells via flow cytometry was increased in the WTC11 iPSC line for D2 (*EOMES*+ mesoderm), D4 (*KDR*+/*PDGFRA*+ CPCs), and D5 (*ISL1*+/*NKX2-5*+ CPCs) reseed conditions in comparison to the CTRL (Figure S8A). For the IMR90-4 iPSC line, the % cTnT cells was increased for D4 (cardiac mesoderm) and D6 (*ISL1*+/*NKX2-5*+ CPCs) reseed conditions in comparison to the CTRL, but it was significantly reduced in the D3 (*EOMES*+ mesoderm) reseed condition in this experiment, which was consistent with the batch variability of the differentiation and reseed treatment (Figure S8E). Across WTC11 and IMR90-4 iPSC lines, reseeding *ISL1*+/*NKX2-5*+ progenitors (D5 WTC11 and D6 IMR90-4), resulted in a significant increase in the MYH7+ single positive CM population by 20–30% (absolute) and a significant reduction in the MYH7+/MYH6+ double-positive CM population by 20–30% (Figures S8B, S8C, S8F–S8G). These changes were also seen in the IMR90-4 iPSC line at the *EOMES*+ mesoderm (D3) and the cardiac mesoderm (D4) stages. The MYH6+ single positive CM population was unchanged across conditions and cell lines (Figures S8D and S8H). Representative images at 20x magnification depict these changes for both cell lines tested (Figures S8I and S8J). These data demonstrate that reseeding has either no effect on myosin heavy chain isoform expression or supports the more mature myosin heavy chain isoform phenotype depending on the stage of differentiation and cell line.

To assess additional CM structural phenotypes, we investigated CM multinucleation and junctional Cx43+ in CMs. For nucleation status, CMs were identified from MYH7+/MYH6+ single and double-positive areas as stained and imaged above, and CM nucleation status was visually determined as being either mononucleated or multinucleated. The percentage of mononucleated CMs for all conditions was between 70 and 80%, which is in a similar range as prior reports of hPSC-CMs.^47^^,^^51^ There were no significant changes in the percentage of mononucleated or multinucleated CMs for any of the reseed conditions in comparison to the control in either cell line tested (Figures S7E and S7F). To examine junctional Cx43 expression in CMs, D23 cardiomyocytes were co-stained for cTnT to identify cardiomyocytes, Cx43 to identify the junctional expression of Cx43 in cTnT+ cardiomyocytes, and DNA (Hoechst 33342). Cx43 exhibited nuclear cross-staining, which complicated quantification. However, imaging at 20x magnification demonstrated that CTRL and all reseeded conditions exhibited junctional Cx43 puncta in cTnT+ CMs. Thus, CTRL and all reseed conditions were capable of forming Cx43+ gap junctions. Representative images are shown for CTRL and one reseed condition in both WTC11 (D5 *ISL1*+/*NKX2-5+* CPCs and IMR90-4 (D3 *EOMES*+ mesoderm) cell lines (Figure S9). Collectively, these data demonstrate that reseeding did not substantially impact contractility, sarcomere structure, multinucleation, or junctional Cx43 expression in CMs in comparison to the control differentiation protocol. However, reseeding *ISL1*+/*NKX2-5*+ CPCs improved the percentage of CMs exhibiting a more mature myosin heavy chain isoform expression profile across cell lines by 20–30% (absolute), while reseeding at other stages of differentiation had cell line-dependent effects.

### *EOMES*+ mesoderm and *ISL1*+/*NKX2-5*+ cardiac progenitors can be cryopreserved to resume differentiation with increased cardiomyocyte purity

**Figure 4:**
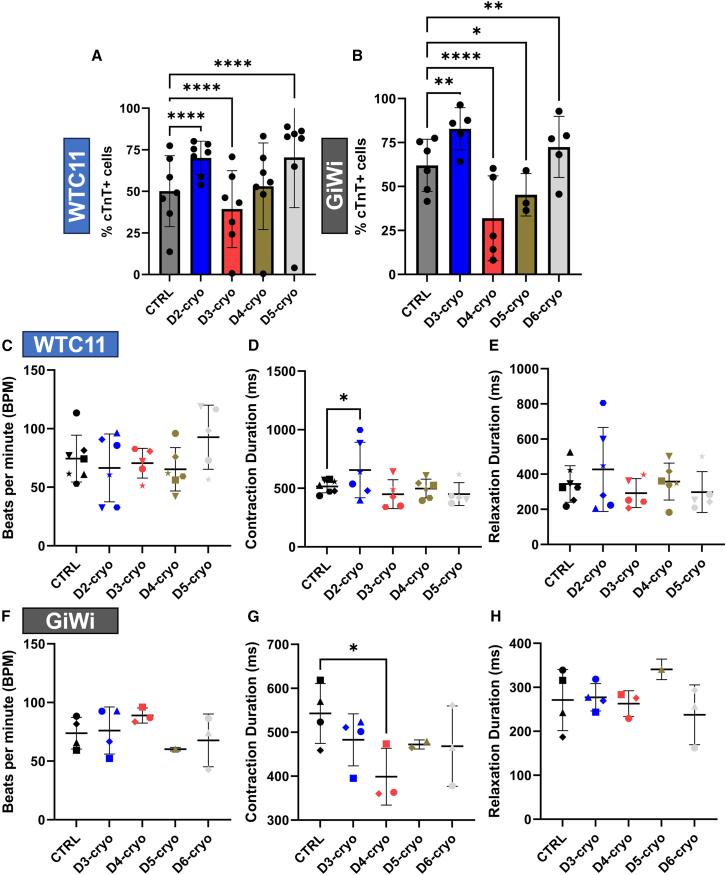
*EOMES*+ mesoderm and *ISL1*+/*NKX2-5*+ cardiac progenitors can be cryopreserved to resume differentiation with increased cardiomyocyte purity (A) Flow cytometry analysis of cTnT expression in hPSC-CMs for control (CTRL) differentiation samples compared to cryopreserved samples at the indicated days in the WTC11 iPSC line. Points represent the mean values of 3–4 technical replicates for 7 independent differentiations. (B) Flow cytometry analysis of cTnT expression in hPSC-CMs for control (CTRL) differentiation samples compared to cryopreserved samples at the indicated days in GiWi cell lines. Points represent the mean values of 2–6 technical replicates for 6 independent differentiations (5 IMR90-4, 1 19-9-11). (C–E) Contractile parameters (beats per minute - C, contraction duration - D, and relaxation duration - E) in CMs differentiated from the WTC11 iPSC line were quantified using MUSCLEMOTION. Points represent the mean values of 1–5 technical replicates for 7 independent differentiations, which are represented by unique symbols. (F–H) Contractile parameters (beats per minute - F, contraction duration - G, and relaxation duration - H) in CMs differentiated from GiWi cell lines were quantified using MUSCLEMOTION. Points represent the mean values of 1–5 technical replicates for 4 independent differentiations, which are represented by unique symbols (3 IMR90-4, 1 19-9-11). All *p*-values from a two-factor linear mixed-effects model with repeated measures testing for a main column effect with a Dunnett’s post-hoc test (except in A, where a full model effect was tested). ∗ for *p* < 0.05, ∗∗ for *p* < 0.01, ∗∗∗ for *p* < 0.001, ∗∗∗∗ for *p* < 0.0001, and ns for *p* > 0.05. All data are represented as mean ± SD.

To extend the practical use of reseeding hPSC-CM progenitors during differentiation, we investigated whether hPSC-CM progenitor populations were amenable to cryopreservation for cell banking purposes. Previous studies have demonstrated that CPCs retain hPSC-CM differentiation capacity after cryopreservation; however, no published studies have demonstrated that earlier hPSC-CM differentiation progenitors are cryopreservable with the ability to efficiently complete CM differentiation.^20^^,^^21^^,^^28^ After detaching and cryopreserving, progenitor cells were thawed and plated at a 1:2.5 ratio by surface area used in non-cryopreserved hPSC-CM differentiation experiments. We observed significant increases in mean CM purity of 20% (absolute) when reseeding cryopreserved progenitors in the WTC11 iPSC line only at the *EOMES*+ mesoderm (D2) and *ISL1*+/*NKX2-5*+ CPC (D5) stages in comparison to the control differentiation (Figures 4A and S10A). In GiWi lines, mean CM purity also increased by 10–20% (absolute) when reseeding cryopreserved progenitors at the corresponding *EOMES*+ mesoderm (D3) and *ISL1*+/*NKX2-5*+ CPC (D6) stages (Figure 4B and S10B). Increases in CM purity for cryopreserved *EOMES*+ mesoderm and *ISL1+/NKX2-5*+ CPCs were consistent with non-cryopreserved reseeding results; however, non-cryopreserved reseeding at the *KDR*+/*PDGFRA*+ CPC stage (D4 WTC11 and D5 GiWi lines) also resulted in an increase in CM purity while cryopreservation and replating at this stage did not increase CM purity (Figures 2A and 2D; Figures 4A and 4B). Ultimately, plating cryopreserved progenitors at the cardiac mesoderm stage (D3 WTC11 and D4 GiWi lines) or at the *KDR*+/*PDGFRA*+ CPC stage (D4 WTC11 and D5 GiWi lines) resulted in reduced CM purity in comparison to control differentiation samples in 3 out of 4 cases (Figures 4B, S10A and S10B). The number of CMs relative to the CTRL in the WTC11 iPSC line was increased after cryopreserving *EOMES+* mesoderm and decreased for *ISL1*+/*NKX2-5*+ CPCs (Figure S10C). However, the number of CMs relative to the CTRL in the GiWi lines was unchanged at both of these stages (Figure S10D). Cryopreservation and replating between these stages significantly reduced the number of CMs relative to CTRL in 3 out of 4 cases. This further supported the idea that cryopreservation and replating at the cardiac mesoderm and *KDR*+/*PDGFRA*+ CPC stages were not beneficial for CM purity and were potentially detrimental to CM number. On the whole, these data demonstrate that cryopreservation and reseeding improved CM purity when performed at the *EOMES*+ mesoderm and *ISL1*+/*NKX2-5*+ CPC stages of hPSC-CM differentiation.

To better understand the differences between cryopreserved and non-cryopreserved reseeding, we compared CM purity and CM number for cryopreserved and non-cryopreserved reseeding between the *EOMES*+ mesoderm and *ISL1*+/*NKX2-5*+ CPC stages across cell lines (Figures S11A–S11D). Cryopreserved reseeding resulted in a lower or similar CM purity as non-cryopreserved reseeding for cardiac mesoderm (D3 WTC11 and D4 GiWi lines) and *KDR*+/*PDGFRA*+ stages (D4 WTC11 and D5 GiWi lines) across cell lines (Figures S11A and S11B). Cryopreserved reseeding also resulted in a lower CM number in comparison to the CTRL differentiation for cardiac mesoderm (D3 WTC11 and D4 GiWi lines) and *KDR*+/*PDGFRA*+ stages (D4 WTC11 and D5 GiWi lines) across cell lines (Figures S11C and S11D). Cryopreserved reseeding at the *EOMES*+ mesoderm (D2 WTC11 and D3 GiWi lines) and *ISL1*+/*NKX2-5*+ CPC stages (D5 WTC11 and D6 GiWi lines) resulted in increased or similar CM purity in comparison to non-cryopreserved reseeding (Figures S11A and S11B). Cryopreserved reseeding resulted in increased or similar CM numbers in comparison to the CTRL at the *EOMES*+ mesoderm stage (D2 WTC11 and D3 GiWi lines) and similar CM numbers in comparison to the CTRL at the *ISL1*+/*NKX2-5*+ CPC stage (D5 WTC11 and D6 GiWi lines). However, the CM number for cryopreserved reseeding trended lower than non-cryopreserved reseeding at these stages (Figures S11C and S11D). These data demonstrate that cryopreserved reseeding supports differentiation at the *EOMES*+ mesoderm (D2 WTC11 and D3 GiWi lines) and *ISL1*+/*NKX2-5*+ CPC stages (D5 WTC11 and D6 GiWi lines) but not at other stages between these time points.

To overcome the limitation of inefficient differentiation at the cardiac mesoderm and *KDR*+/*PDGFRA*+ CPC stages, we attempted to improve CM differentiation after progenitor cryopreservation by introducing treatments to aid in cell recovery from cryopreservation. These included a higher seeding ratio by surface area of 1:1 (as opposed to 1:2.5) and the addition of ROCK inhibition with 5 μM Y-27632 on the day of thawing and plating (Figures S11E and S11F). These treatments had no obvious benefit for improving CM purity for any of the cryopreserved conditions in any of the cell lines and did not appear to improve cell recovery.

To test whether cryopreservation significantly impacted contractile function, we acquired videos and analyzed contractile parameters using MUSCLEMOTION.^37^ Similar to non-cryopreserved reseeding, the cryopreservation of cardiac progenitors did not have any conserved impacts on beat rate, contraction duration, or relaxation duration in comparison to the control differentiation protocol between WTC11 (Figures 4C–4E) and GiWi cell lines (Figures 4F–4H). Contraction duration was significantly decreased when reseeding at Day 4 in GiWi cell lines and significantly increased at Day 2 for the WTC11 iPSC line; however, no other parameters were changed between any of the other conditions. There were also a few statistically significant changes in contractile parameters when plating cryopreserved progenitors with increased density or ROCK inhibition (Figure S12). Taken together, these data are consistent with previous findings for non-cryopreserved reseeding (Figures 3A–3F), indicating that reseeding did not significantly impact contractile function regardless of whether cells are reseeded before or after cryopreservation.

### *EOMES*+ mesoderm and *ISL1*+/*NKX2-5*+ cardiac progenitors can be cryopreserved and differentiated on defined extracellular matrix proteins

After establishing that *EOMES+* mesoderm and *ISL1*+/*NKX2-5*+ CPC stage hPSC-CM progenitors can be efficiently recovered after cryopreservation and differentiate to CMs with increased efficiency, we explored whether thawing and plating these cryopreserved progenitors on defined extracellular matrix (ECM) proteins could further improve CM purity. The standard hPSC-CM differentiation protocol is performed on Matrigel, which was selected because it supports hPSC expansion and permits hPSC-CM differentiation. Cryopreservation of hPSC-CM progenitors permits hPSC expansion and early differentiation on Matrigel, followed by plating cells after cryopreservation on a different substrate, which may be better for late-stage hPSC-CM differentiation or downstream applications. We identified 3 ECM proteins commonly utilized in hPSC-CM differentiation and culture, fibronectin (FN), laminin-111 (LAM1), and vitronectin (VTN), to compare against thawing and plating on Matrigel (MG), the standard matrix used for hPSC-CM differentiation (and all prior reseeding experiments).^31^^,^^32^^,^^33^^,^^34^ *EOMES*+ mesoderm progenitors and *ISL1*+/*NKX2-5*+ CPCs plated from cryopreservation on vitronectin and fibronectin exhibited a similar confluent monolayer appearance to cells thawed and plated on Matrigel and to non-cryopreserved cells reseeded on Matrigel following recovery on D16 (Figures S13A–S13D, Figure S3). Cells thawed and plated on laminin-111 displayed a unique cluster-like morphology on D16 (Figure S13B). Additionally, plating cryopreserved *EOMES*+ mesoderm progenitors and *ISL1*+/*NKX2-5*+ CPCs resulted in a similar or improved CM purity compared to non-cryopreserved reseeding and similar or decreased CM number in comparison to non-cryopreserved reseeding and CTRL differentiation samples (Figures S13E–S13H).

**Figure 5:**
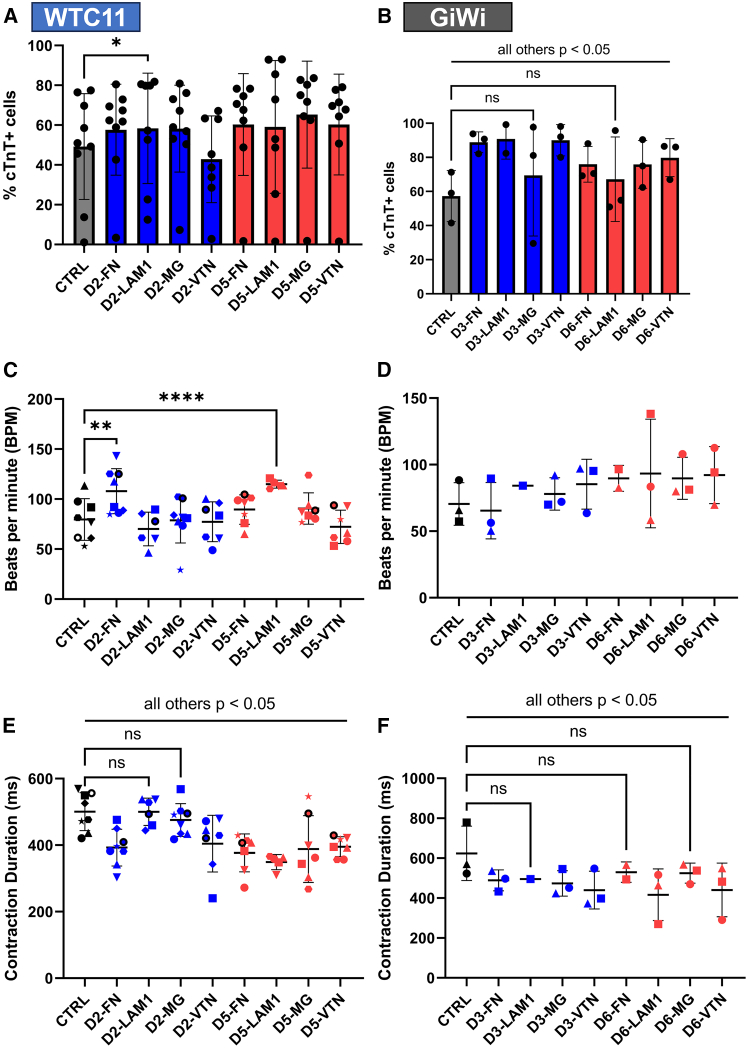
*EOMES*+ mesoderm and *ISL1*+/*NKX2-5*+ cardiac progenitors can be cryopreserved and differentiated on defined extracellular matrix proteins (A) Flow cytometry analysis of cTnT expression in hPSC-CMs for control (CTRL) differentiation samples compared to cryopreserved samples plated onto Fibronectin (FN), Laminin-111 (LAM1), Matrigel (MG), or Vitronectin (VTN) at either D2 or D5 from the WTC11 iPSC line. Points represent the mean values of 2–4 technical replicates for 9 independent differentiations. (B) Flow cytometry analysis of cTnT expression in hPSC-CMs for control (CTRL) differentiation samples compared to cryopreserved samples plated onto Fibronectin (FN), Laminin-111 (LAM1), Matrigel (MG), or Vitronectin (VTN) at either D3 or D6 from GiWi cell lines. Points represent the mean values of 3–4 technical replicates for 3 independent differentiations (2 IMR90-4, 1 19-9-11). (C and E) Contractile parameters (beats per minute – **C** and contraction duration – **E** in CMs differentiated from the WTC11 iPSC line quantified using MUSCLEMOTION. Points represent the mean values of 1–4 technical replicates for 8 independent differentiations, which are represented by unique symbols. (D and F) Contractile parameters (beats per minute – **D** and contraction duration – **F** in CMs differentiated from GiWi cell lines quantified using MUSCLEMOTION. Points represent the mean values of 1–4 technical replicates for 3 independent differentiations, which are represented by unique symbols (2 IMR90-4, 1 19-9-11). All *p*-values from two-factor linear mixed-effects model with repeated measures testing for a main column effect with a Dunnett’s post-hoc test. ∗ for *p* < 0.05, ∗∗ for *p* < 0.01, ∗∗∗ for *p* < 0.001, ∗∗∗∗ for *p* < 0.0001, and ns for *p* > 0.05. All data are represented as mean ± SD.

Across cell lines, plating cryopreserved *EOMES*+ mesoderm progenitors and *ISL1*+/*NKX2-5*+ CPCs on fibronectin increased CM purity by 8–30% (absolute), which was similar to Matrigel reseed controls (Figures 5A and 5B, S14C and S14D). Moreover, thawing and plating on fibronectin at both stages resulted in the most consistent cell recovery from cryopreservation with equivalent CM numbers relative to the control differentiation samples (Figures S14A and S14B). Thawing and plating on laminin-111 significantly improved mean CM purity by 10–30% (absolute) only at the *EOMES*+ mesoderm stage across cell lines (Figures 5A and 5B, S14C and S14D); however, it also significantly reduced relative CM number (Figures S14A and S14B). Lastly, thawing and plating on vitronectin improved mean CM purity by 10–20% (absolute) across cell lines only at the *ISL1*+/*NKX2-5*+ CPC stage (Figures 5A and 5B, Figure S14C and S14D), while also not impacting relative CM number (Figures S14A and S14B). These results demonstrate that hPSC-CM differentiation can be resumed on several different defined ECM proteins following *EOMES*+ mesoderm and *ISL1*+/*NKX2-5*+ CPC cryopreservation. Additionally, these data highlight potential stage-specific effects of extracellular matrix proteins during hPSC-CM differentiation.

To evaluate differences in contractile function in terminal CMs resulting from *EOMES*+ mesoderm and *ISL1*+/*NKX2-5*+ CPCs reseeded after cryopreservation on defined ECMs, we again employed MUSCLEMOTION to interrogate beat rate, contraction duration, and relaxation duration.^37^ Spontaneous beat rate was largely unaffected in most of the conditions across cell lines; however, *EOMES*+ mesoderm stage thawing and plating on fibronectin and *ISL1*+/*NKX2-5*+ CPC stage thawing and plating on laminin-111 significantly increased beat rate in the WTC11 iPSC line (Figures 5C and 5D). Additionally, contraction duration was significantly reduced in many of the replated ECM conditions across both cell lines (Figures 5E and 5F); however, there were fewer conserved differences in relaxation duration between the control differentiation protocol and cryopreserved conditions across both cell lines (Figures S14E and S14F). Given that there were few differences in contractile parameters in previous non-cryopreserved reseed (Figure 3) and cryopreserved plating on Matrigel (Figure 4) experiments, the observed contractile differences are most likely due to altering the ECM composition. Moreover, our findings are consistent with previous reports demonstrating that the culture substrate can influence the contractile parameters of hPSC-CMs.^47^

## Discussion

This study demonstrates that harvesting and reseeding hPSC-CM progenitors, namely *EOMES*+ mesoderm progenitors (D2 WTC11 and D3 GiWi) and *ISL1*+/*NKX2-5*+ CPCs (D5 WTC11 and D6 GiWi), at a lower cell density increases terminal CM purity by roughly 10–20% (absolute) during small molecule-based hPSC-CM differentiation. Moreover, this process did not substantially impact CM number, contractile function, sarcomere length, CM multinucleation, or junctional expression of Cx43. However, reseeding *ISL1*+/*NKX2-5*+ CPCs consistently increased the percentage of single positive MYH7+ CMs by 20–30% (absolute) and concurrently decreased the percentage of double-positive MYH7+/MYH6+ CMs by 20–30% (absolute) across cell lines. These data represent a modest early maturation shift toward a more mature myosin heavy chain isoform expression profile. This early maturation shift was also seen when reseeding several other progenitor stages in the IMR90-4 iPSC line.

Although it is known that the initial cell seeding density is a critical factor for commitment to the CM lineage during hPSC-CM differentiation, reports investigating the effects of cell density during monolayer CM differentiation have been limited.^8^^,^^32^^,^^35^^,^^36^^,^^52^ Buikema et al. showed that removal of cell-cell contacts in combination with transient canonical Wnt activation using CHIR99021 can expand Day 12 hPSC-CMs up to 250-fold without affecting resultant CM function; however, this treatment occurred after commitment to the CM lineage.^45^^,^^46^ Additionally, Le and colleagues demonstrated that Transwell co-culture of CM differentiations with high and low initial cell seeding density can improve the CM differentiation efficiency of low density cultures by promoting the transition from the mesoderm to CPC stage of differentiation via paracrine Wnt signaling inhibition.^53^ Furthermore, using a process modeling approach to identify predictors of CM differentiation success in 3D bioreactor suspension culture, Williams et al. suggested that cell density may play a role in predicting terminal CM content between D3-D7 of differentiation in addition to initial cell seeding density.^11^

Few studies have investigated reseeding at a lower density during monolayer hPSC-CM differentiation; however, other reports have demonstrated that reseeding, also referred to as replating, during mesoderm differentiation to other lineages from hPSCs can improve differentiation efficiency.^54^^,^^55^^,^^56^ Moreover, the dissociation and reaggregation of hPSC-derived mesoderm and CPC 3D spheroids have been reported to be important for optimizing CM production from hPSCs, potentially through the activation of endogenous Wnt inhibitors when dissociation and reaggregation is performed at the CPC stage.^57^^,^^58^ This study demonstrates that reseeding hPSC-CM progenitors at a lower cell density between the *EOMES*+ mesoderm and *ISL1*+/*NKX2-5*+ stages can improve CM differentiation efficiency. While we demonstrated that *ISL1*+/*NKX2-5*+ CPC reseeding can increase the expression of CM marker gene *TNNT2* and reduce the expression of canonical CPC marker genes *NKX2-5* and *MEF2C* immediately after reseeding, the exact mechanism of CM enrichment remains unknown. Several studies have demonstrated that paracrine signaling plays a role in the early stages of hPSC-CM differentiation, including signaling pathways such as TGF-β, Wnt, and nucleotide signaling.^35^^,^^52^^,^^53^^,^^59^ Thus, it is plausible that the alteration of paracrine signaling may play a role in the observed effects of reseeding at a lower cell density during progenitor hPSC-CM stages. However, it is also possible that certain cardiomyocyte-fated progenitors exhibit a survival, attachment, or proliferative advantage during the reseeding process. Collectively, prior literature and this study underscore the importance of cell density both at the onset and during hPSC differentiation to CMs and other mesodermal lineages.

This study also establishes that only cryopreserved *EOMES*+ mesoderm progenitors and *ISL1*+/*NKX2-5*+ CPCs are able to differentiate to CMs with significantly improved efficiency upon reseeding at a lower density, without affecting contractile properties and with cell line-dependent effects on CM number. While prior reports have reported that CPCs maintain hPSC-CM differentiation capacity following cryopreservation, our study demonstrates that *EOMES*+ mesoderm progenitors can also be cryopreserved while retaining the capacity to proficiently differentiate to functional hPSC-CMs.^20^^,^^28^^,^^29^^,^^30^ Vahdat and colleagues previously demonstrated that mesoderm progenitors (termed cardiogenic mesodermal cells or CMCs), could be efficiently cryopreserved and differentiated to several mesodermal lineages; however, although cardiomyocyte differentiation yielded cTnT+ cells, these cells failed to spontaneously contract in culture, which is a crucial hallmark of hPSC-CM differentiation and function.^21^ Given the demonstrated ability to improve CM purity and generate functional hPSC-CMs from cryopreserved *EOMES*+ mesoderm progenitors and *ISL1*+/*NKX2-5*+ CPCs, this work will enable the production of large-scale purity-validated hPSC-CMs for research and biomedical applications. Because of the ability to validate CM purity from parallel cryovials of the same batch, this approach makes it possible to investigate *EOMES*+ mesoderm progenitors and *ISL1*+/*NKX2-5*+ CPCs predicted to differentiate to CMs with high efficiency to understand molecular features that contribute to differentiation success. Moreover, cryopreservation permits precise experimental timeline synchronization to facilitate rigorous large-scale screens across multiple cell lines and batches to improve CM differentiation starting as early as the *EOMES*+ mesoderm stage of differentiation.

Several recent reports have demonstrated that fresh and cryopreserved hPSC-derived CPCs may have their own therapeutic potential for myocardial infarction or ischemic heart failure in large animal models.^60^^,^^61^ Moreover, hPSC-derived CPCs generated under current good manufacturing practice (cGMP) regulations have demonstrated safety following transplantation in humans with ischemic heart failure.^62^^,^^63^ While we did not generate or cryopreserve hPSC-derived *EOMES*+ mesoderm progenitors or *ISL1*+/*NKX2-5*+ CPCs following cGMP practices, this will be important for clinical applications of these cells. For example, CryoStor CS10, which is a cryopreservation medium manufactured under cGMP standards, has been successfully used to cryopreserve hPSCs, CPCs, and hPSC-CMs with similar cell recovery to FBS-containing cryopreservation medium.^22^^,^^60^^,^^64^ CPCs have several potential advantages as a cell source for cardiac repair, including the ability to generate multiple cardiac cell types and a higher proliferation potential than hPSC-CMs. Raval et al. generated *NKX2-5+/PDGFRA*+ D6 committed CPCs and demonstrated that they were able to engraft at the site of infarcted swine myocardium and generate cardiomyocytes and endothelial cells in as few as 2 weeks with improvements in left ventricular volume reduction and contractile reserve.^60^ Yap et al. similarly generated *NKX2-5*+/*PDGFRA*+ D7 committed CPCs and demonstrated the presence of cardiomyocytes, endothelial cells, and fibroblasts as early as 4 weeks after transplantation in infarcted swine myocardium. Furthermore, they demonstrated that transplanted committed CPCs led to a reduction in infarct size and an improvement in left ventricular ejection fraction that persisted for 12 weeks, which was the final follow-up time point.^61^ On the whole, these data demonstrate that hPSC-CM progenitors are prime candidates for basic science and translational research investigations.

ECM proteins are known to play critical roles in development, but are typically understudied in comparison to simpler, soluble signaling differentiation perturbations. Reseeding cryopreserved *EOMES*+ mesoderm progenitors and *ISL1*+/*NKX2-5*+ CPCs on fibronectin, laminin-111, and vitronectin also supported hPSC-CM differentiation, demonstrating a potential application of this method to further investigate the role of ECM during late-stage CM development from progenitor populations. For this set of experiments, fibronectin was selected given its recently established role in mesoderm formation and cardiac differentiation *in vitro.*^31^ Vitronectin was selected given its known ability to support the entire hPSC-CM differentiation protocol *in vitro.*^32^^,^^33^^,^^65^ Lastly, laminin-111 was selected because Matrigel consists of roughly ∼60% laminin of which laminin-111 is the predominant isoform.^34^ Future studies could explore additional defined ECM components as well as engineered synthetic hydrogels to influence CM differentiation. In addition to ECM applications, lifting and reseeding hPSC-CM progenitors sets the stage for the future exploration of progenitor expansion to increase CM number as well as the long-term maintenance of self-renewing progenitor populations, which have been done in other hPSC-derived cell types.^21^^,^^66^^,^^67^^,^^68^^,^^69^

Methods to improve the purity of hPSC-CM differentiation and reduce batch-to-batch and line-to-line variability have been described, but more work is necessary to optimize a universal protocol that reproducibly and cost-effectively generates >70% CMs. Previous studies have investigated a number of protocol adaptations to increase CM purity, including dual Wnt inhibition, PI3K inhibition, MAPK inhibition, cell cycle inhibition, ascorbate supplementation, nicotinamide supplementation, as well as other treatments.^9^^,^^36^^,^^70^^,^^71^^,^^72^^,^^73^ However, no studies have systematically tested combinations of these protocol innovations to enhance hPSC-CM differentiation success. By demonstrating that *EOMES*+ mesoderm progenitors and *ISL1*+/*NKX2-5*+ CPCs can be cryopreserved and reseeded with increased CM purity, we establish an additional method to improve CM purity and allow for intermediate cell banking of key developmental progenitors capable of differentiating to CMs and other off-target lineages. Using these cryopreserved progenitors, future studies could perform stage-specific screens of small molecule libraries or literature-curated lists to investigate the impact on hPSC-CM differentiation purity. This work and future hPSC-CM differentiation protocol improvements will promote robust, high-purity hPSC-CM differentiation that is less reliant on the prior optimization of CHIR99021 and initial cell seeding density for each hPSC line. Ultimately, this work will help to facilitate the production of highly pure CMs for cell therapy, studies of development and disease, and drug toxicity and discovery applications.

### Limitations of the study

Our study was limited to assessing the effects of reseeding and cryopreservation during small molecule-based monolayer hPSC-CM differentiation and did not assess applicability to growth factor-based or 3D hPSC-CM differentiation protocols. Researchers should verify that reseeding and cryopreservation function similarly for hPSC-CM differentiation protocol variations and additional cell lines. Appropriate timing may differ across protocols and cell lines, and researchers trying to adapt this protocol should factor in the molecular markers reported here as well as the timing of Wnt inhibition if it is present. Although reseeding and cryopreservation did not have substantial effects on cardiomyocyte quality, a full demonstration of the effects of reseeding on cardiomyocyte quality would require more investigation, including experiments such as transcriptomics, action potential characterization, and contractile force measurement. Results from thawing cryopreserved *EOMES*+ mesoderm progenitors and *ISL1*+/*NKX2-5*+ CPCs onto Matrigel and defined ECM proteins varied slightly from prior experiments. Matrigel reseeding (used in prior experiments) did not consistently improve purity (Figure 5). For this set of experiments, ECMs were coated for only 1 h, whereas in prior experiments, Matrigel plates were typically coated overnight. In certain cases, the effects of reseeding and cryopreservation exhibited batch-to-batch and line-to-line variation. The rationale for how reseeding improves purity is not yet understood, nor is the mechanistic basis for batch-to-batch and line-to-line variation.

## Resource availability

### Lead contact

Requests for further information and resources should be directed to and will be fulfilled by the lead contact, Sean P. Palecek (sppalecek@wisc.edu).

### Materials availability

All primer sequences are published in Table S1. All other materials used in this study are publicly available.

### Data and code availability

All of the data and statistical tests used to generate each figure have been deposited on Mendeley (data.mendeley.com) and are publicly available as of the date of publication at Mendeley Data: https://doi.org/10.17632/dzxcw45vp6.1. This article does not report original code. Any additional information required to reanalyze the data reported in this article is available from the lead contact upon request.

## Acknowledgments

The authors thank Fathima Shabnam for her assistance and input on the project. The authors thank Alex Pinto, a University of Wisconsin-Madison Biostatistician from the Department of Biostatistics and Medical Informatics, for his assistance and input on the project. The authors thank the University of Wisconsin Carbone Cancer Center Flow Cytometry Laboratory, supported by P30 CA014520, for use of its facilities and services. This work was supported by research grants awarded by the 10.13039/100000050National Heart, Lung, and Blood Institute (NHLBI, 5R01HL148059 and 1F30HL173988-01), the 10.13039/100000002National Institutes of Health Medical Scientist Training Program (NIH, T32 GM140935), the National Institute on Aging Biology of Aging and Age Related Diseases Training Program (NIA, T32 AG000213), the National Institute for General Medical Sciences Biotechnology Training Program (NIH, T32 GM135066), and the National Science Foundation Center for Cell Manufacturing Technologies Engineering Research Center (NSF; CMaT EEC-1648035). The graphical abstract BioRender: https://BioRender.com/u40o768 and the schematic in Figure 1A BioRender: https://BioRender.com/i87d305 were created in BioRender.

## Declaration of interests

The authors declare no competing interests.

## Author contributions

Conceptualization, investigation, resources, supervision, and project administration, A.K.F. and A.D.S.; data curation and writing – original draft and visualization, A.K.F.; methodology, validation, software, and formal analysis, A.K.F., A.D.S., C.J.P., and X.Z.; writing – review and editing, all authors; funding acquisition, S.P.P.

## STAR★Methods

### Key resources table

**Table:** 

REAGENT or RESOURCE	SOURCE	IDENTIFIER
**Antibodies**		

Mouse IgG1 anti-cTnT	ThermoFisher	Cat#MA512960; RRID: AB_11000742
Goat anti-mouse IgG Alexa Fluor 488	ThermoFisher	Cat#A21121; RRID: AB_2535764
Mouse IgG2b anti-MF20	DSHB	N/A; RRID: AB_2147781
Mouse IgG1 anti-alpha-actinin	Sigma-Aldrich	Cat#A7811; RRID: AB_476766
Goat anti-mouse IgG2b Alexa Fluor 488	ThermoFisher	Cat#21141; RRID: AB_2535778
Goat anti-mouse IgG1 Alexa Fluor 647	ThermoFisher	Cat#21240; RRID: AB_2535809
Rabbit IgG anti-MYH7	R&D Systems	Cat#MAB90961100; RRID: AB_3659321
Mouse IgG1 anti-MYH6	R&D Systems	Cat#MAB8979; RRID: AB_3659283
Goat anti-rabbit IgG Alexa Fluor 488	ThermoFisher	Cat#A11008; RRID: AB_143165
Rabbit IgG anti-Cx43	Cell Signaling Technology	Cat#3512; RRID: AB_2294590
Goat anti-rabbit IgG Alexa Fluor 647	ThermoFisher	Cat#A21246; RRID: AB_2535814

**Chemicals, peptides, and recombinant proteins**		

Growth Factor Reduced Matrigel	Corning	Cat#354263
Versene	Life Technologies	Cat#15040066
Accutase	Innovative Cell Technology	Cat#AT104
Y-27632	Tocris	Cat#1254
CHIR99021	Selleckchem	Cat#S1263
IWP2	Tocris	Cat#3533
Fetal bovine serum	R&D Systems	Cat#S12450
Dimethyl Sulfoxide (DMSO)	Sigma-Aldrich	Cat#D2650
Cultrex Mouse Laminin I, Pathclear	R&D Systems	Cat#340001002
Human Plasma Fibronectin Purified Protein	Sigma-Aldrich	Cat#FC010
Animal-Free Recombinant Human Vitronectin	PeproTech	Cat#AF14009
Paraformaldehyde	Electron Microscopy Sciences	Cat#15710-S
Bovine Serum Albumin (BSA)	ThermoFisher	Cat#BP1600
Triton X-100	ThermoFisher	Cat#BP151
Hoechst 33342	Invitrogen	Cat#H3570
Trizol Reagent	ThermoFisher	Cat#15596018
RNaseOUT Recombinant Ribonuclease Inhibitor	Life Technologies	Cat#10777-019
Oligo dT(20) primers	Life Technologies	Cat#18418020
PowerUp SYBR Green Master Mix for qPCR	ThermoFisher	Cat#25780

**Experimental models: Cell lines**		

Human: IMR90-4 hiPSC line (female, viral transfection)	Yu et al., 2007^74^	N/A
Human: 19-9-11 hiPSC line (male, non-integrating)	Yu et al., 2009^75^	N/A
Human: WTC11 hiPSC line (male, non-integrating)	Kreitzer et al.^76^	N/A
Human: H9 hESC line (female, embryonic)	Thomson et al.^77^	N/A

**Software and algorithms**		

MUSCLEMOTION (version 1.1)	Sala et al.^37^	https://github.com/l-sala/MUSCLEMOTION
Sotatool	Stein et al.^50^	https://github.com/steinjm/SotaTool
ImageJ	Schneider et al.^78^	https://imagej.nih.gov/ij/
GraphPad Prism (version 9.4.1)	N/A	https://graphpad.com
BioRender	N/A	https://biorender.com
Nikon Instruments Software Elements (version 5.30.06)	N/A	https://www.microscope.healthcare.nikon.com/products/software/nis-elements
MetaboAnalyst (version 6.0)	Pang et al.^79^	https://www.metaboanalyst.ca/MetaboAnalyst/ModuleView.xhtml

**Oligonucleotides**		

Primers for RT-qPCR: See Table S1.	This paper	N/A

**Critical commercial assays**		

Zymo RNA Clean & Concentrator-25	Zymo Research	Cat#R1018
Qiagen Omniscript RT Kit	Qiagen	Cat#205113

**Deposited data**		

Mendeley dataset	data.mendeley.com	Mendeley Data: https://doi.org/10.17632/dzxcw45vp6.1

**Other**		

mTeSR1 medium	STEMCELL Technologies	Cat#85850
DMEM/F12 medium	ThermoFisher	Cat#11330032
RPMI1640	Life Technologies	Cat#11875119
B27 minus insulin supplement	Life Technologies	Cat#0050129SA
B27 plus insulin supplement	Life Technologies	Cat#17504044
Mr. Frosty Freezing Container	ThermoFisher	Cat#51000001
Dulbecco’s Phosphate Buffered Saline (DPBS)	ThermoFisher	Cat#14190144
C-Slide Chamber Slide	NanoEnTek - VWR	Cat#76627-758

### Experimental model and study participant details

The human, female IMR90-4 iPSC line was used in this study. The human, male 19-9-11 iPSC line was used in this study. The human, male WTC11 iPSC line was used in this study. The human, female H9 ESC line was used in this study. The maintenance of these 4 hPSC lines is described below. The influence of cell line sex was not directly reported in this study, but all data are included in supplemental data tables. All hPSC lines were authenticated using STR profiling at WiCell in Madison, Wisconsin. Routine testing for mycoplasma contamination was performed every 6 to 12 months.

### Method details

#### Human pluripotent stem cell maintenance

hPSCs (iPSCs [IMR90-4, 19-9-11, WTC11] and ESCs [H9]) were cultured as previously described.^32^^,^^74^^,^^75^^,^^76^^,^^77^ In short, hPSCs were cultured on Growth Factor Reduced Matrigel (0.5 mg/plate or 11 μg/cm^2^; Corning - 354263) resuspended in DMEM/F12 (ThermoFisher - 11330032) on 6-well plates (Corning COSTAR – 07-200-82). Cells were maintained in mTeSR1 medium (STEMCELL Technologies – 85850) and passaged every 3-5 days at 60-80% confluency with Versene incubation for 6-10 minutes (Life Technologies - 15040066) at 37°C. Cells were maintained in a cell culture incubator (Sanyo MCO-18AC; 37°C, 5% CO2, 95% RH).

#### hPSC-cardiomyocyte differentiation

hPSCs were differentiated using the GiWi protocol previously developed in our laboratory.^32^^,^^35^ hPSCs were lifted and singularized with Accutase (Innovative Cell Technology - AT104) for 5 minutes. After cell detachment, the Accutase cell solution was diluted 1:1 v/v with DMEM/F12 to diminish enzymatic activity. Cells were manually counted and centrifuged at 200G for 5 minutes. Cells were seeded for hPSC-CM differentiation on Day -2 using cell densities expected to work well for each cell line and incubated at room temperature for 30 minutes prior to transfer to a cell culture incubator. For all cell lines, media for Day -2 was mTeSR1 medium with 5 μM Y-27632 (Tocris - 1254) and media for Day -1 was mTeSR1 medium. For all cell lines, differentiation was initiated on Day 0 in RPMI1640 (Life Technologies - 11875119) + 2% v/v B27 minus insulin (B27-; Life Technologies - 0050129SA) using CHIR99021 (Selleckchem - S1263) at a concentration between 6-12 μM expected to work well for each cell line. After Day 0, differentiation protocol timing is cell line dependent as previously reported (Figure 1A).^38^^,^^39^ For the WTC11 cell line, media was changed on Day 2 with B27- and 5 μM IWP2 (Tocris - 3533) and on Day 4 with B27-. On Days 6, 8, 10 and every 3 days after, media was changed with RPMI1640 + B27 supplement (B27+, Life Technologies - 17504044) until Day 15-19 collection. For all GiWi cell lines (IMR90-4, H9, 19-9-11), media was changed on Days 1 and 5 with B27- and on Day 3 with 1:1 v/v B27-:conditioned medium with 5 μM IWP2. Starting on Day 7, media was changed every 3 days with B27+ until Day 15-19 collection. Cells were only collected for flow cytometry and other experiments if the cells appeared alive and uncontaminated. In some cases, although minimal or no spontaneous beating was observed, cells were collected for further analysis.

#### hPSC-CM progenitor reseeding

Early hPSC-CM progenitors (Days 2-5 for WTC11 cell line and Days 3-6 for GiWi cell lines) were gently lifted during differentiation using cold Accutase for 5 minutes at 37°C followed by trituration with a P1000 2-3 times. After 5 minutes of cold Accutase treatment, the cells were still loosely attached to the plate, and P1000 trituration 2-3 times removed them from the plate as a mixture of single cells and small clusters. The Accutase cell solution was diluted 1:4 v/v with DMEM/F12 and centrifuged at 200G for 5 minutes. Cells were resuspended in RPMI1640 + 2% v/v B27 minus insulin (standard differentiation medium - B27-) and reseeded at a lower density onto Matrigel-coated wells at a ratio of 1:2.5 by surface area (1 lifted well to 2.5 reseeded wells of equivalent surface area) unless otherwise indicated. 5 μM IWP2 was added for WTC11 progenitors reseeded on D2 or GiWi cell line progenitors (IMR90-4, H9, 19-9-11) reseeded on D3 following the standard hPSC-CM differentiation protocol (above).

#### Cryopreservation and thawing of hPSC-CM progenitors

hPSC-CM progenitors (Days 2-6) were lifted and pelleted as above and subsequently resuspended in progenitor freezing medium containing 6:3:1 v/v/v differentiation media (B27-), fetal bovine serum (FBS, R&D Systems – S12450), and dimethyl sulfoxide (DMSO, Sigma-Aldrich – D2650) using a Mr. Frosty Freezing Container (ThermoFisher - 51000001) filled with isopropanol as reported previously in our laboratory for hPSCs and other hPSC-derived cell types.^80^^,^^81^^,^^82^ In brief, filled cryovials were placed in a Mr. Frosty which was transferred to a -80°C freezer overnight. The subsequent day, vials were transferred to liquid nitrogen for long term cryo-storage.

For cryopreservation experiments, frozen cryovials of hPSC-CM progenitors were thawed rapidly in a 37°C water bath for 1-3 minutes while moving the cryovials back and forth until a small ice crystal remained. Subsequently, Dulbecco’s Phosphate Buffered Saline (DPBS) was added dropwise to thawed hPSC-CM progenitors in progenitor freezing medium to avoid osmotic shock prior to transferring the cell solution to 5 mL DPBS. The DPBS cell solution was centrifuged at 200G for 5 minutes prior to resuspending the cells in the standard differentiation medium for reseeding at a 1:2.5 ratio by surface area unless otherwise indicated.

#### Defined extracellular matrix coatings

Published ECM coating concentrations in the context of hPSC-CM differentiation were used to inform ranges for pilot studies evaluating progenitor cell attachment for Laminin I, Fibronectin, and Vitronectin.^31^^,^^33^^,^^65^ For all subsequent experiments, Cultrex Mouse Laminin I, Pathclear (R&D Systems – 340001002) was resuspended in DMEM/F12 and plates were coated at 5 μg/cm^2^. Human Plasma Fibronectin Purified Protein (Sigma-Aldrich – FC010) was resuspended in DMEM/F12 and plates were coated at 2.5 μg/cm^2^. Animal-Free Recombinant Human Vitronectin (PeproTech - AF14009) was resuspended in DPBS (ThermoFisher - 14190144) and plates were coated at 3.8 μg/cm^2^. All defined extracellular matrix coatings were performed the same day that cells were plated and allowed to incubate at 37°C in a cell culture incubator for at least 1 hour prior to use.

#### Flow cytometry analysis of cardiac troponin T

Flow cytometry was performed at the end of hPSC-CM differentiation to determine cardiomyocyte purity using cardiac troponin T (cTnT). Cells from each individual well were rinsed with DPBS and then detached and singularized with Accutase for 10-20 minutes prior to dilution with 1:4 v/v with DMEM/F12 and centrifugation at 200G for 5 minutes. Fixation was performed with 1% (v/v) paraformaldehyde (Electron Microscopy Sciences - 15710-S) in DPBS for 20 minutes prior to centrifugation at 200G for 5 minutes and resuspension in 90% (v/v) ice-cold methanol. Cells were stored at -20°C until flow cytometry sample preparation. 90% (vol/vol) methanol was washed from each sample with Flow Buffer 1 (FB1 = 0.5% w/v Bovine Serum Albumin [BSA, ThermoFisher- BP1600] in DPBS). Following centrifugation at 300G for 5 minutes, cells were resuspended in Flow Buffer 2 (FB2 = FB1 + 0.1% (v/v) Triton X-100 (ThermoFisher – BP151)) and incubated overnight at 4°C with 1:1000 msIgG1-anti-cTnT primary antibody (ThermoFisher – MA512960). The primary antibody solution was washed from each sample with FB1 followed by centrifugation at 300G for 5 minutes. Cells were incubated for 30-60 minutes at room temperature in the dark with 1:1000 AF488-anti-msIgG secondary antibody (ThermoFisher - A21121) in FB2. The secondary antibody solution was washed from each sample with FB1 followed by centrifugation at 300G for 5 minutes. Finally, wells were resuspended in FB1 prior to analysis on a C6 Plus Accuri or ThermoFisher Attune flow cytometer. Undifferentiated hPSC negative control samples were prepared identically and analyzed in parallel for gating purposes. Cell, single cell, and cTnT gating were performed in either the BD Accuri C6 Plus software or FlowJo (Figures S2A and S2B).

#### Cell counting for relative cardiomyocyte number

Cell counts were obtained from flow cytometry at the end of hPSC-CM differentiation to determine the number of CMs relative to CTRL. For flow cytometry counts, an equivalent amount of each differentiation well was analyzed and the number of CMs in the cTnT+ gate was obtained. To determine relative CM number, the number of CMs in each sample within a differentiation was divided by the average number of CMs in the control condition. For samples reseeded at a ratio of 1:2.5 by surface area, the number of CMs was multiplied by 2.5 to account for the split ratio. Relative CM number was also determined through manual counts for a limited number of non-cryopreserved reseed differentiations (Figures S5C and S5D). After individual wells were fixed and stored in 90% methanol at -20°C prior to flow cytometry, 10 μL was used for manual cell counting. For each differentiation, cell counts were determined visually or using a cell cytometer (Invitrogen Countess II FL). For visual cell counts, 10 μL of the 90% methanol/cell solution was loaded into a reusable hemocytometer. For cell cytometer cell counts, the 90% methanol/cell solution was combined with Trypan Blue 1:1 v/v, and 10 μL was loaded into disposable slides (C-Slide Chamber Slide, NanoEnTek - VWR 76627-758). Relative CM number was calculated as described above.

#### Cell counting on day of progenitor reseeding

Cell counts on the day of differentiation seeding (Day -2) and the day of non-cryopreserved progenitor reseeding (Days 2-5 for WTC11 and Days 3-6 for GiWi cell lines) were obtained to determine the number of cells present on each day and the number of cells used for reseeding experiments (number of cells present/2.5 split ratio). For cell counts, 10 μL of the differentiation medium/cell solution was loaded into a reusable hemocytometer and visually counted (Figure S7).

#### Video-based contractility analysis

Phase-contrast video acquisition was performed at 20x magnification on a Nikon Ti2e with an ORCA-Flash4.0 camera (Hamamatsu C13440-20CU). Videos of cardiomyocyte contraction were acquired at 50 frames (FPS) per second for 20 seconds once in each well. To accommodate 50 FPS acquisition, videos were recorded using 12 bit-depth and 4x4 binning using the NIS-Elements Software. ND2 files were converted to AVI or TIF prior to video-based contractility analysis using the open-source ImageJ macro MUSCLEMOTION (version 1.1) using the default software parameters and the recommended speedWindow between 2 to 5.^37^^,^^78^ The measured and calculated speed tracings were visually inspected for each video to ensure reliable contractile parameter quantification as outlined in the user manual. For samples with unreliable matching between the measured and calculated speed tracings due to low signal or high noise, the data were excluded from further analysis. Peak-to-peak time (ms) was used to calculate beats per minute (BPM) using the following equation: 60∗1000/peak-to-peak time.

#### Immunocytochemistry for cardiomyocyte phenotype analysis

On Day 16, cells were rinsed with DPBS and lifted with Accutase for 10-20 minutes prior to dilution with 1:4 v/v with DMEM/F12 and centrifugation at 200G for 5 minutes. Cells were replated at a lower density (90,000-240,000 cells/cm^2^) in B27+ on Matrigel-coated 24- or 96-well imaging plates (Ibidi – 82426, 89626) and allowed to recover for 7 days with B27+ media changes on the day after replating (Day 17) and 3 days after (Day 20). Cells were rinsed with DPBS and fixed for 20-30 minutes with 1% (v/v) paraformaldehyde at room temperature. Another DPBS rinse was performed prior to blocking with 0.5-1% (w/v) BSA in DPBS and storage at 4°C.

After removal from storage, wells were rinsed with DPBS and incubated with primary antibodies overnight in the dark at 4°C on a shaker in FB2. The primary antibody solution was rinsed with DPBS, and wells were incubated with secondary antibodies for 60 minutes at room temperature in the dark in FB2. The secondary antibody solution was rinsed with DPBS, and wells were incubated with 1:5000 (2μg/ml) Hoechst 33342 (Invitrogen - H3570) in DPBS for 5 minutes. Wells were rinsed with DPBS twice and filled with DPBS for imaging (microscope above, equipped with an Aura III light engine (Lumencor 80-10306)).

For sarcomere length analysis, primary antibodies were used at the following dilutions: 1:25 ms-IgG2b-anti-MF20 (DSHB) and 1:1000 ms-IgG1-anti-alpha-actinin (Sigma-Aldrich – A7811). Secondary antibodies were used at the following dilutions: 1:1000 AF488-anti-msIgG2b secondary antibody (ThermoFisher – A21141) and 1:1000 AF647-anti-msIgG1 secondary antibody (ThermoFisher – A21240). Cardiomyocytes were identified from MF20 staining and sarcomeres were identified from alpha-actinin staining. Sarcomere lengths were manually quantified at 60x magnification using ImageJ across four Z lines (or bands) in 10 sarcomeres for each image as previously described.^49^ Sarcomere lengths were also automatically quantified at 60x (9.09 pixels/μm) and 40x (6.25 pixels/μm) magnification using SotaTool with the following analysis parameters: minimum sarcomere length – 1.0 μm, maximum sarcomere length – 2.5μm, 4x4 segmentation, offset – 4 μm, and default values for all other parameters.^50^

For myosin heavy chain isoform analysis, primary antibodies were used at the following dilutions: 1:100 rb-IgG-anti-MYH7 (R&D Systems – MAB90961100) and 1:100 ms-IgG1-anti-MYH6 (R&D Systems – MAB8979). Secondary antibodies were used at the following dilutions: 1:1000 AF488-anti-rbIgG secondary antibody (ThermoFisher – A11008) and 1:1000 AF647-anti-msIgG1 secondary antibody (ThermoFisher – A21240). At 20x magnification, beta-myosin heavy chain positive cardiomyocytes were identified from MYH7 staining, and alpha-myosin heavy chain positive cardiomyocytes were identified from MYH6 staining. The total area of cardiomyocytes identified from MYH7 and MYH6 staining was determined with a binary threshold and quantified using Nikon Imaging Software (NIS) Elements version 5.30.06. Hoechst positive cardiomyocyte nuclei were identified within the MYH7 and MYH6 positive regions using a binary threshold. The cardiomyocyte nuclei area within double positive and single positive MYH7 and MYH6 regions was then used to determine the percentage of cardiomyocytes in the following populations: MYH7+/MYH6+, MYH7+ only, and MYH6+ only.

For Cx43 analysis, primary antibodies were used at the following dilutions: 1:500 rb-IgG-anti-Cx43 (Cell Signaling Technology – 3512) and 1:1000 ms-IgG1-anti-cTnT (ThermoFisher – MA512960). Secondary antibodies were used at the following dilutions: 1:1000 AF647-anti-rbIgG secondary antibody (ThermoFisher – A21246) and 1:1000 AF488-anti-msIgG1 secondary antibody (ThermoFisher – A21120). Presence of junctional Cx43 in cTnT+ cardiomyocytes was visualized at 20x magnification in NIS Elements (version 5.30.06).

For cardiomyocyte multinucleation analysis, MYH7 and MYH6 were used to identify cardiomyocytes, and Hoechst was used to identify nuclei within cardiomyocytes at 20x magnification. The number of mononucleated cardiomyocytes was manually counted for the entire image, followed by the manual count of multinucleated cardiomyocytes. For images with dense cell clustering, nucleation status was determined within two circular regions of interest (ROIs). The nucleation status of each image was counted twice, and the values were averaged to determine the percentage of mono- and multinucleated cardiomyocytes. At least 15 cardiomyocytes were counted per image and at least 83.5 cardiomyocytes were counted per condition across 4 image replicates.

#### RNA isolation and reverse transcription quantitative polymerase chain reaction (RT-qPCR)

RNA samples were collected from 1-well of a 12-well plate as previously described.^38^ Wells were rinsed once with DPBS before a 1-minute incubation with 500 μL of cold Trizol reagent (ThermoFisher - 15596018). Samples were transferred into 1.5mL microcentrifuge tubes, snap-frozen in liquid nitrogen, and stored at -80°C. After thawing, samples underwent a tri-phasic extraction to collect RNA, which was then concentrated and purified with Zymo RNA Clean & Concentrator-24 columns (Zymo Research – R1018), following the kit instructions. A NanoDrop Spectrophotometer (ThermoFisher – ND2000c) was used to determine the concentration of the extracted RNA, which was stored at -80°C prior to reverse transcription.

Once thawed, manufacturer’s instructions were followed on the Qiagen Omniscript RT Kit (Qiagen – 205113) to reverse transcribe 2 μg of RNA into cDNA, using RNaseOUT (Life Technologies – 10777-019) and Oligo dT(20) primers (Life Technologies – 18418020) as described previously.^38^ Each reaction contained 10 ng of sample cDNA, 12.5 μL PowerUp SYBR (ThermoFisher – A25780), 0.125 μL of appropriate primers (IDT, 100 μm), and nuclease-free water to create a total volume of 25 μL/reaction. qPCR reactions underwent 40 thermocycles between 95°C (15 s) and 60°C (60 s) on an AriaMx Real-Time PCR system (Agilent Technologies – G8830A). Melt curve examination confirmed the presence of a single amplicon product for all reactions. *ZNF384*, *DDB1*, and *EDF1* were included as references genes as described previously.^38^ *EDF1* was selected as the reference gene as it exhibited the lowest coefficient of variation across plates and samples. Statistics were performed on normally distributed delta delta Ct values using *EDF1* as the reference gene and using the CTRL condition or the Day 0 condition as the reference sample. Undetected Ct values were replaced with maximum detected Ct value plus 3 cycles for the gene of interest within that differentiation or the maximum cycle number of 40, whichever value was lower. Graphical representations used 2ˆ(-delta delta Ct values) to depict relative gene expression.

#### qPCR primer design and validation

Primers were designed using the NCBI Primer-BLAST online tool and sequences ordered from IDT as described previously.^38^ Amplicon size specificity was confirmed by agarose gel electrophoresis. Primers that generated one amplicon band at the correct size in a positive control sample (10 ng of WTC11 cDNA) were then validated for primer efficiency using qPCR with a four-point standard curve over a 1:5 dilution series ([cDNA] = 10,2, 0.4, 0.08 ng/reaction) and a no template control ([cDNA] = 0 ng/reaction]. Primers exhibiting no amplification of the no template control, and primer efficiencies between 90% and 110% were released for further use (see key resources table for primer details). *KDR*, *PDGFRA*, and *TBX5* primers were designed for this study while all other primers have been previously published by our laboratory.^38^^,^^39^

### Quantification and statistical analyses

Statistical analyses were performed in GraphPad Prism (version 9.4.1). P-values were either listed as numbers or indicated with following symbols ∗ for p < 0.05, ∗∗ for p < 0.01, ∗∗∗ for p < 0.001, ∗∗∗∗ for P < 0.0001, and ns for p > 0.05. P-values were calculated using a paired t-test, a simple linear regression, an ordinary one-way ANOVA, a two-way ANOVA, or a two-factor linear mixed-effects model with repeated measures with a Dunnett’s or Tukey’s post-hoc for multiple comparisons as indicated. All error bars indicate standard deviation. Graphs were created with GraphPad Prism and schematics were created with BioRender.com. RT-qPCR heatmaps were created using the one-factor Statistical Analysis module in MetaboAnalyst (version 6.0).^79^

## Supplemental information

Document S1. Figures S1–S14

Table S1. RT-qPCR primers used for measuring gene expression, related to Figures 1, S2, and S4

Table S2. Data for reseeding at the cardiac progenitor stage of hPSC-CM differentiation, related to Figures 1, S1, and S2 A) Fig 1B data. B) Fig 1C data. C) Fig 1D data. D) Fig 1E data. E) Fig S1B data. F) Fig S1C data. G) Fig S1D data. H) Fig S1F data. I) Fig S1G data. J) Fig S1H data. K) Fig S2A data. L) Fig S2C data. M) Fig S2D data. N) Fig S2E data. O) Fig S2F data.

Table S3. Data for reseeding early progenitors of hPSC-CM differentiation, related to Figures 2, S4, S5, and S6 A) Fig 2A data. B) Fig 2B data. C) Fig 2C data. D) Fig 2D data. E) Fig 2E data. F) Fig 2F data. G) Fig S4B data. H) Fig S4D data. I) Fig S5A data. J) Fig S5B data. K) Fig S5C data. L) Fig S5D data. M) Fig S5E data. N) Fig S5F data. O) Fig S6A data. P) Fig S6B data. Q) Fig S6C data. R) Fig S6D data. S) Fig S6E data. T) Fig S6F data.

Table S4. Data for phenotypic impacts of reseeding early progenitors of hPSC-CM differentiation, related to Figures 3, S7, and S8 A) Fig 3A data. B) Fig 3B data. C) Fig 3C data. D) Fig 3D data. E) Fig 3E data. F) Fig 3F data. G) Fig 3G data. H) Fig 3H data. I) Fig S7A data. J) Fig S7B data. K) Fig S7C data. L) Fig S7D data. M) Fig S7E data. N) Fig S7F data. O) Fig S8A data. P) Fig S8B data. Q) Fig S8C data. R) Fig S8D data. S) Fig S8E data. T) Fig S8F data. U) Fig S8G data. V) Fig S8H data.

Table S5. Data for cryopreservation and reseeding of early progenitors of hPSC-CM differentiation, related to Figures 4, S10, S11, and S12 A) Fig 4A data. B) Fig 4B data. C) Fig 4C data. D) Fig 4D data. E) Fig 4E data. F) Fig 4F data. G) Fig 4G data. H) Fig 4H data. I) Fig S10A data. J) Fig S10B data. K) Fig S10C data. L) Fig S11A data. M) Fig S11B data. N) Fig S11C data. O) Fig S11D data. P) Fig S11E data. Q) Fig S11F data. R) Fig S12A data. S) Fig S12B data. T) Fig S12C data. U) Fig S12D data. V) Fig S12E data. W) Fig S12F data.

Table S6. Data for cryopreservation and reseeding of EOMES+ mesoderm and ISL1/NKX2-5 cardiac progenitors on defined extracellular matrix proteins, related to Figures 5, S13, and S14 A) Fig 5A data. B) Fig 5B data. C) Fig 5C data. D) Fig 5D data. E) Fig 5E data. F) Fig 5F data. G) Fig S13E data. H) Fig S13F data. I) Fig S13G data. J) Fig S13H data. K) Fig S14A data. L) Fig S14B data. M) Fig S14C data. N) Fig S14D data. O) Fig S14E data. P) Fig S14F data.
