# Supplementary material for: Enhancing human pluripotent stem cell differentiation to cardiomyocytes through cardiac progenitor reseeding and cryopreservation
Source: iScience. 2025 Apr 16;28(5):112452. doi: 10.1016/j.isci.2025.112452 (PMC12124670; doi:10.1016/j.isci.2025.112452)
Supplement: Document S1. Figures S1–S14 [file mmc1.pdf]

iScience, Volume *28*

Supplemental information

Enhancing human pluripotent stem cell differentiation to cardiomyocytes through cardiac progenitor reseeding and cryopreservation

Austin K. Feeney, Aaron D. Simmons, Claire J. Peplinski, Xiaotian Zhang, and Sean P. Palecek

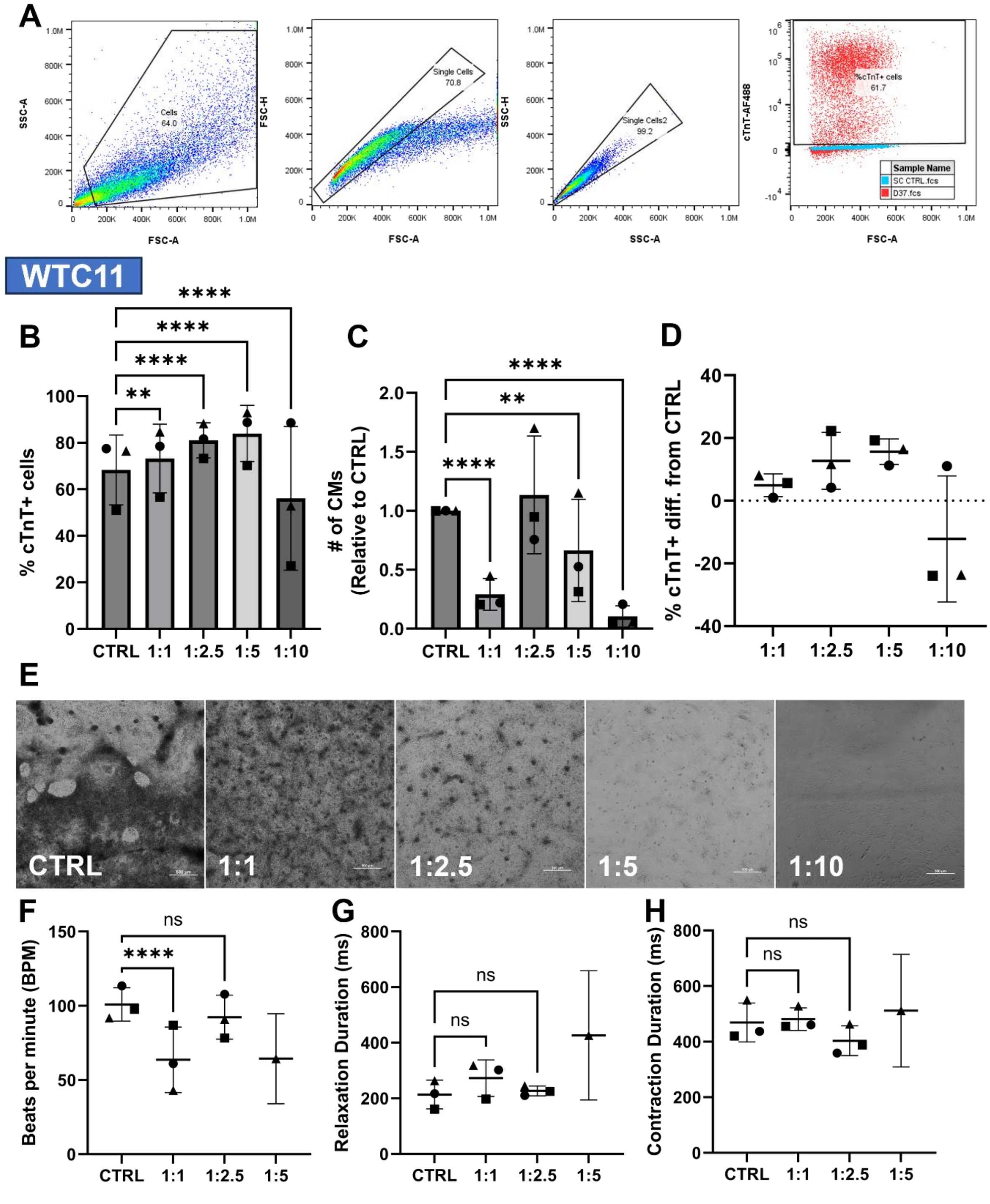

A
SSC-A
FSC-A
Cells
64.0
FSC-H
Single Cells
70.8
SSC-H
Single Cells2
99.2
cTnT-AF488
%cTnT+ cells
61.7
Sample Name
SC CTRL.fcs
D37.fcs
WTC11
B
% cTnT+ cells
CTRL 1:1 1:2.5 1:5 1:10
**
****
****
****
C
# of CMs
(Relative to CTRL)
****
**
****
CTRL 1:1 1:2.5 1:5 1:10
D
% cTnT+ diff. from CTRL
1:1 1:2.5 1:5 1:10
E
CTRL
1:1
1:2.5
1:5
1:10
F
Beats per minute (BPM)
ns
****
CTRL 1:1 1:2.5 1:5
G
Relaxation Duration (ms)
ns
ns
CTRL 1:1 1:2.5 1:5
H
Contraction Duration (ms)
ns
ns
CTRL 1:1 1:2.5 1:5

Figure S1 (Related to Figure 1). Flow cytometry gating strategy. The effects of cryopreserved CPC reseeding at multiple surface area ratios on CM purity, CM number, cell recovery, and contraction properties. A) Flow cytometry dot plots demonstrating representative gating for cells (FSC-A versus SSC-A), single cells (FSC-A versus FSC-H and SSC-A versus SSC-H), and cTnT expression in the IMR90-4 iPSC line. cTnT gating shown in a stem cell control (SC CTRL, blue) and a D16 hPSC-CM sample (sample D37, red). **B)** Flow cytometry analysis of cTnT expression in D16 hPSC-CMs for control (CTRL) differentiation samples compared to CPC reseeding at 1:1, 1:2.5, 1:5, and 1:10 ratios by surface area following cryopreservation. Points represent the mean values of 3-4 technical replicates for 3 independent differentiations represented by unique shapes in the WTC11 iPSC line. **C)** The number of CMs relative to CTRL differentiation samples. Points represent the mean values of 3-4 technical replicates for 3 independent differentiations represented by unique symbols in the WTC11 iPSC line. **D)** Absolute difference in the percentage of cTnT+ cells for the indicated split compared to the CTRL of the same differentiation. Unique symbols represent independent WTC11 iPSC differentiations. **E)** Representative phase contrast images of CPC reseed samples at D16 for 1:1, 1:2.5, 1:5, and 1:10 ratios by surface area and corresponding CTRL samples from an hPSC-CM differentiation in the WTC11 iPSC line (D16 cTnT purity for CTRL = 77.5%, for 1:1 CPC Reseed = 78.4%, for 1:2.5 CPC Reseed = 81.7%, for 1:5 CPC Reseed = 88.7%, and for 1:10 CPC Reseed = 88.5%). All images are at 4x magnification with scale bars = 500 µm. **F-H)** Contractile parameters (beats per minute – **F**, relaxation duration – **G**, and contraction duration - **H**) quantified using MUSCLEMOTION. Points represent the mean values of 3-4 technical replicates for 3 independent differentiations represented by unique symbols in the WTC11 iPSC line. Statistics excluded 1:5 and 1:10 reseed conditions as only one and zero differentiations were accurately quantified respectively. All P-values from two-factor linear mixed-effects model with repeated measures testing for full model effects with a Dunnett's post-hoc test. All data are represented as mean ± SD.

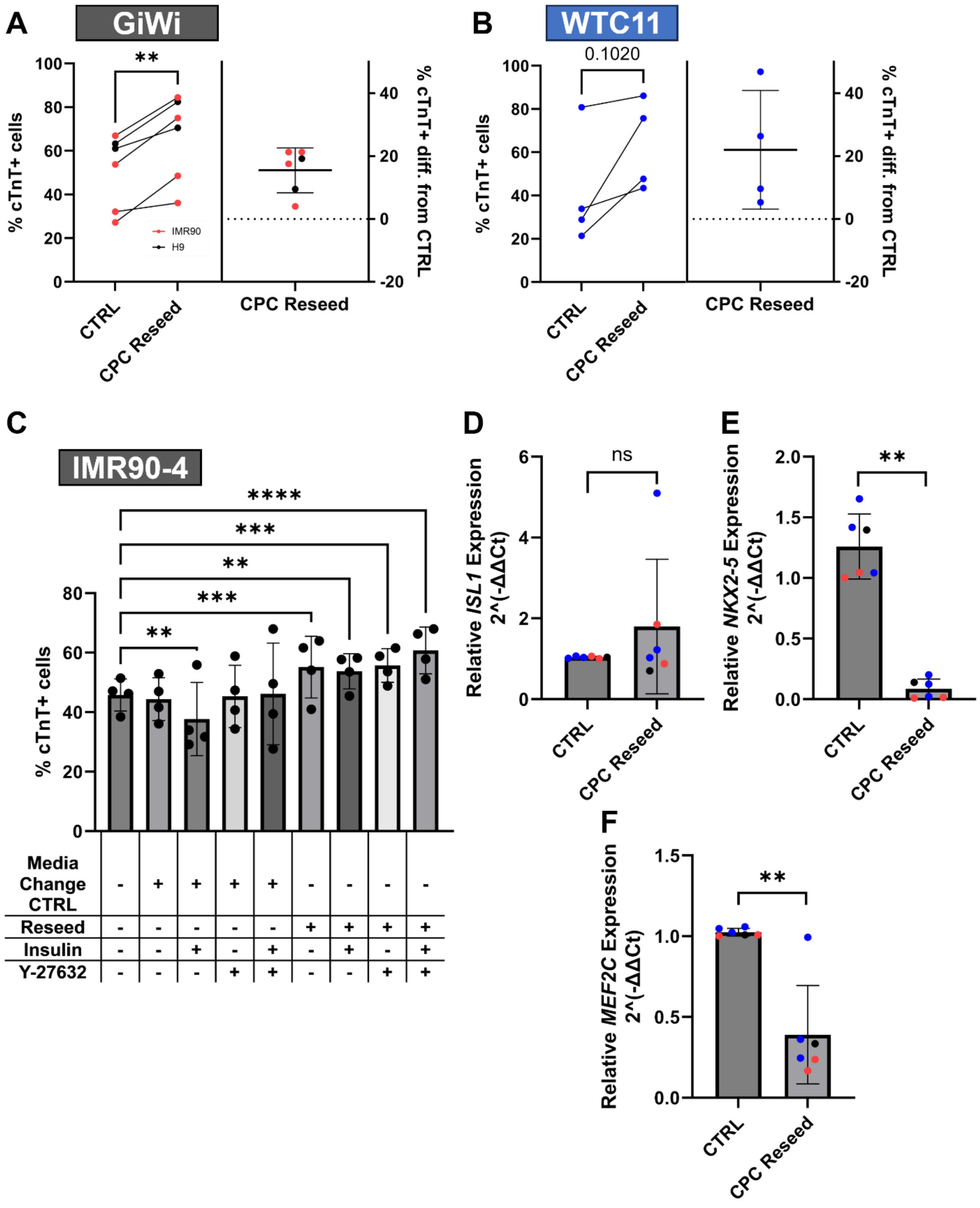

A
GiWi
B
WTC11
% cTnT+ cells
% cTnT+ diff. from CTRL
**
0.1020
IMR90
H9
CTRL
CPC Reseed
C
IMR90-4
****
***
**
***
**
Media Change CTRL
Reseed
Insulin
Y-27632
D
ns
Relative ISL1 Expression 2^(-ΔΔCt)
E
**
Relative NKX2-5 Expression 2^(-ΔΔCt)
F
**
Relative MEF2C Expression 2^(-ΔΔCt)

Figure S2 (Related to Figure 1). CPC reseeding results for WTC11 and GiWi lines. The effects of media composition on CPC reseed and the effects of CPC reseed on the gene expression of CPC markers. A-B) Flow cytometry analysis of cTnT expression in D16 hPSC-CMs for control (CTRL) differentiation samples compared to CPC reseed samples in **A)** GiWi cell lines (4 IMR90-4, 2 H9) and **B)** WTC11 iPSC lines. Points (left portion of A and B) represent the mean values of 3-4 technical replicates for 6 independent differentiations in the GiWi lines and 4 independent differentiations in the WTC11 iPSC line represented by distinct colors. Points (right portion of A and B) represent the absolute difference in the percentage of cTnT+ cells in the CPC reseed condition and the CTRL condition for each differentiation replicate across cell lines represented by distinct colors. P-values from paired t-test. **C)** Flow cytometry analysis of cTnT expression in D16 hPSC-CMs for control (CTRL, column 1) GiWi differentiation samples compared to media change CTRL samples without reseeding and CPC reseed samples with changing media conditions (± insulin and ± Y-27632 at 5 μM). Points represent the mean values of 3-4 technical replicates for 4 independent differentiations in the IMR90-4 iPSC line. P-values from two-factor linear mixed-effects model with repeated measures testing for full model effects with a Dunnett's post-hoc test. RT-qPCR of relative **D)** *ISL1*, **E)** *NKX2.5*, and **F)** *MEF2C* expression ($2^{[-\Delta\Delta Ct]}$) for CTRL differentiation samples compared to CPC reseeded samples one to two days after CPC reseed recovery (D6 for WTC11, D7 for H9, D7-8 for IMR90-4). Points represent the mean values of 2-3 differentiation well replicates for 6 independent differentiation represented by distinct colors (blue = 3 WTC11, red = 2 IMR90-4, black = 1 H9). P-values from paired t-test. All data are represented as mean ± SD.

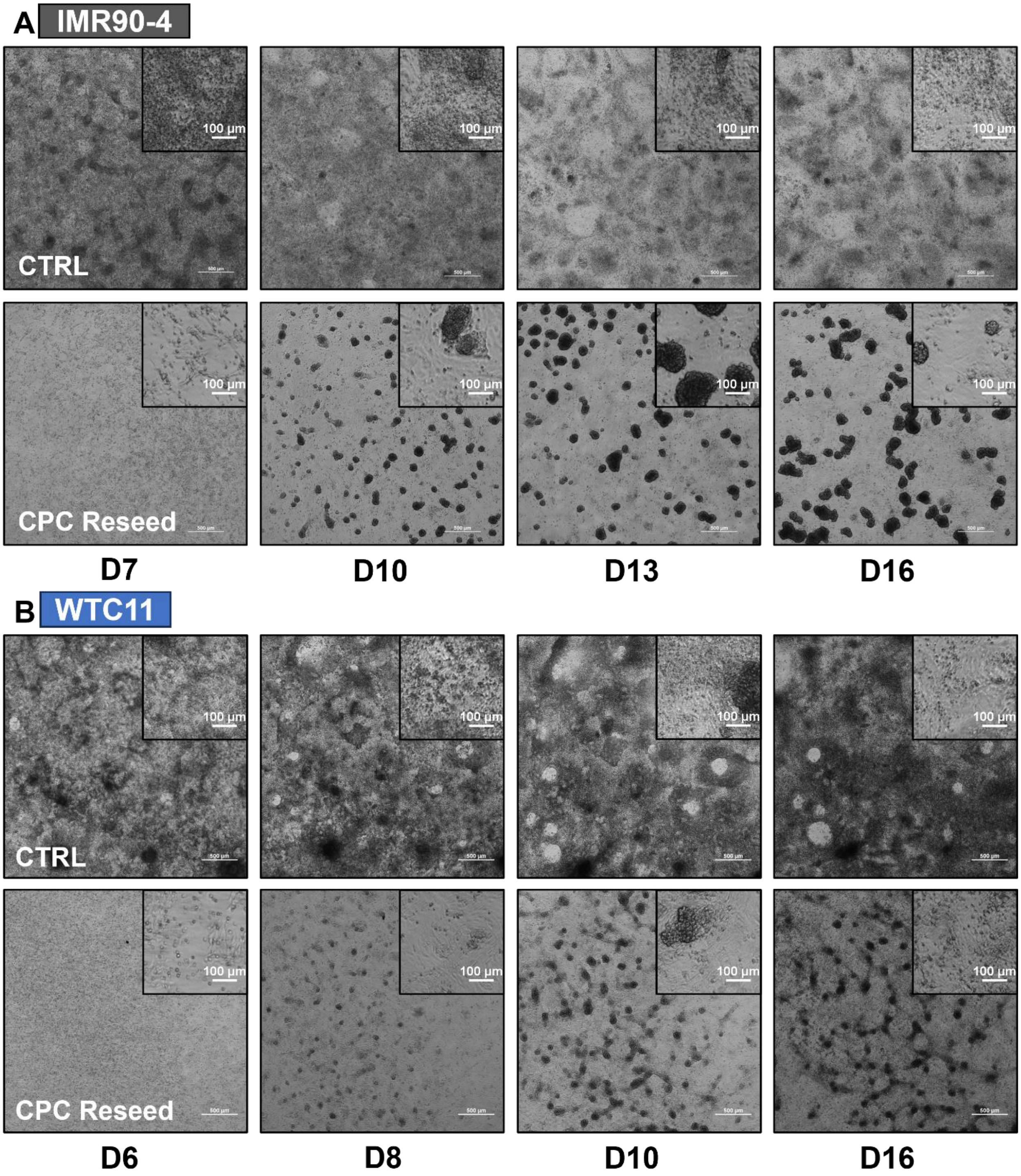
A
IMR90-4
100 µm
CTRL
CPC Reseed
D7
D10
D13
D16
B
WTC11
D6
D8
D10
D16

Figure S3 (Related to Figure 1). Cell recovery after CPC reseed. A) Representative phase contrast images of CPC Reseed samples 1 day, 4 days, 7 days, and 10 days after reseeding and corresponding CTRL samples from an hPSC-CM differentiation using the IMR90-4 iPSC line (D16 cTnT purity for CTRL = 39.5% and for CPC Reseed = 66.5%). **B)** Representative phase contrast images of CPC Reseed samples 1 day, 3 days, 5 days, and 11 days after reseeding and corresponding CTRL samples from an hPSC-CM differentiation using the WTC11 iPSC line (D16 cTnT purity for CTRL = 77.5% and for CPC Reseed = 83.9%). All images are at 4x magnification with scale bars = 500 μm. Insets with scale bars = 100 μm.

A WTC11

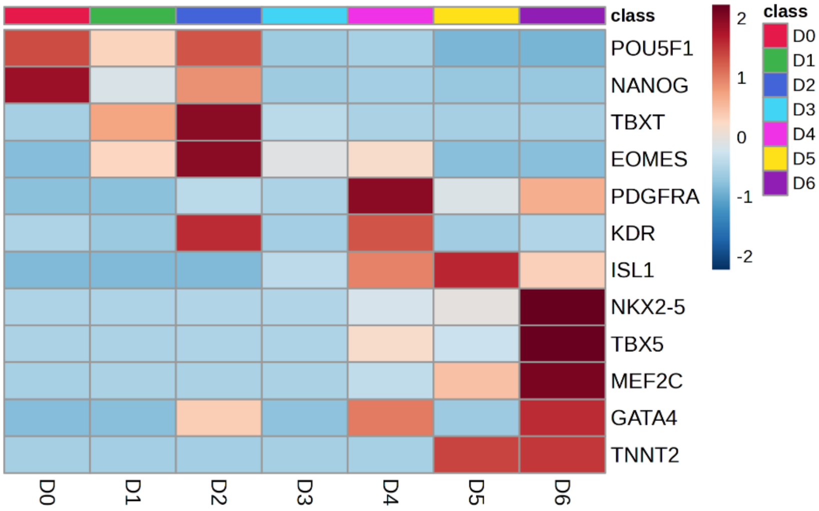

B

WTC11						
**Gene**	**D1**	**D2**	**D3**	**D4**	**D5**	**D6**
**POU5F1**	ns	ns	**	**	****	****
**NANOG**	ns	ns	***	**	****	****
**TBXT**	****	****	****	****	***	*
**EOMES**	****	****	****	****	***	***
**PDGFRA**	***	****	****	****	****	****
**KDR**	ns	*	ns	*	ns	ns
**ISL1**	ns	ns	****	****	****	****
**NKX2-5**	ns	ns	ns	***	***	****
**TBX5**	ns	****	***	****	****	****
**MEF2C**	ns	*	ns	***	****	****
**GATA4**	***	****	****	****	****	****
**TNNT2**	ns	***	ns	ns	****	****

C GiWi

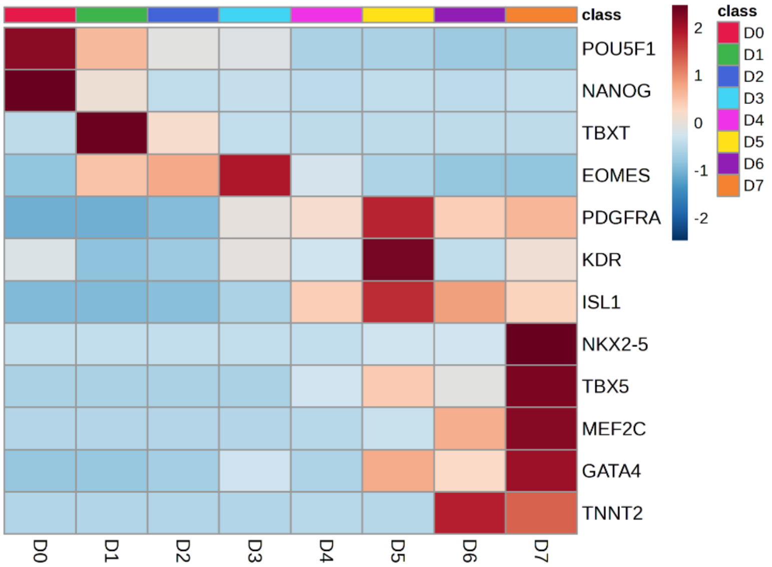

D

GiWi lines							
**Gene**	**D1**	**D2**	**D3**	**D4**	**D5**	**D6**	**D7**
**POU5F1**	**	****	****	****	****	****	****
**NANOG**	ns	***	***	****	****	****	***
**TBXT**	****	****	***	ns	ns	ns	ns
**EOMES**	****	****	****	****	****	****	**
**PDGFRA**	ns	***	****	****	****	****	****
**KDR**	***	*	ns	ns	*	ns	ns
**ISL1**	*	****	****	****	****	****	****
**NKX2-5**	ns	ns	ns	ns	*	**	***
**TBX5**	ns	ns	ns	ns	**	**	**
**MEF2C**	*	ns	ns	ns	***	****	****
**GATA4**	***	****	****	****	****	****	****
**TNNT2**	ns	*	ns	ns	ns	****	****

Figure S4 (Related to Figure 2). Heatmaps of temporal gene expression of undifferentiated, mesoderm, cardiac progenitor, and cardiomyocyte markers for WTC11 and GiWi lines. A) Relative gene expression ($2^{-\Delta\Delta Ct}$) normalized to D0 for differentiation stage markers (z-score normalized for each marker) from D0-D6 in the WTC11 iPSC line. Heatmap values represent 3 independent CTRL WTC11 differentiations with D16 cTnT purity greater than 68%. **B)** P-values from two-way ANOVA testing for main column effect with a Dunnett's post-hoc test. For each differentiation stage marker, D1-D6 ΔΔCt values were compared to D0 ΔΔCt values for the WTC11 iPSC line. **C)** Relative gene expression ($2^{-\Delta\Delta Ct}$) normalized to D0 for differentiation stage markers (z-score normalized for each marker) from D0-D7 in GiWi lines (2 H9, 1 IMR90-4). Heatmap values represent 3 independent CTRL GiWi differentiations from D0-D6 and 2 independent CTRL GiWi differentiations (1 H9, 1 IMR90-4) for D7 with D16 cTnT purity greater than 61%. **D)** P-values from two-way ANOVA testing for a main column effect with a Dunnett's post-hoc test. For each differentiation stage marker, D1-D7 ΔΔCt values were compared to D0 ΔΔCt values for GiWi lines (2 H9, 1 IMR90-4). Heatmap data for a specific gene and time point are represented as mean z-score values for each gene.

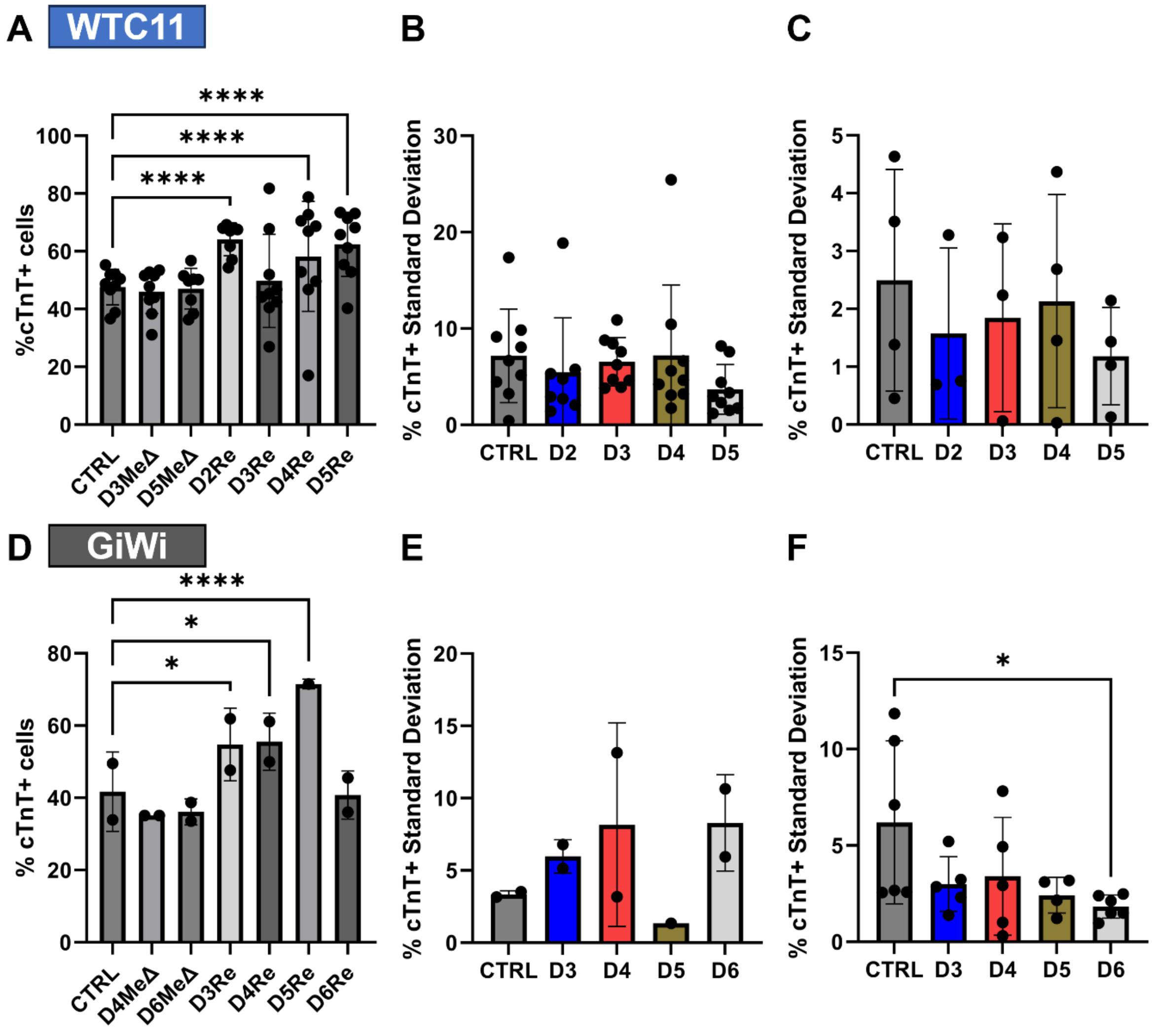
A
WTC11
B
C
D
GiWi
E
F
%cTnT+ cells
% cTnT+ Standard Deviation
% cTnT+ cells
****
*
CTRL
D3MeΔ
D5MeΔ
D2Re
D3Re
D4Re
D5Re
D4MeΔ
D6MeΔ
D6Re
D2
D3
D4
D5
D6

Figure S5 (Related to Figure 2). The effects of media change without reseeding and within differentiation standard deviation for CM purity for pooled and unpooled reseed samples. A) Flow cytometry analysis of cTnT expression in hPSC-CMs for control (CTRL) differentiation samples compared to reseed samples (Re) and non-standard media change samples (MeΔ) at the indicated days in the WTC11 iPSC line. Points represent the mean values of 3-6 technical replicates for 9 independent differentiations. **B)** Within differentiation standard deviation in flow cytometry cTnT expression in hPSC-CMs for control (CTRL) differentiation samples compared to unpooled reseed samples. Points represent the standard deviation values of 3-6 technical replicates for 9 independent differentiations in the WTC11 iPSC line. **C)** Within differentiation standard deviation in flow cytometry cTnT expression in hPSC-CMs for control (CTRL) differentiation samples compared to pooled reseed samples. Points represent the standard deviation values of 3-4 technical replicates for 4 independent differentiations in the WTC11 iPSC line. **D)** Flow cytometry analysis of cTnT expression in hPSC-CMs for control (CTRL) differentiation samples compared to reseed samples (Re) and non-standard media change samples (MeΔ) at the indicated days in the IMR90-4 iPSC line. Points represent the mean values of 3-4 technical replicates for 2 independent differentiations. **E)** Within differentiation standard deviation in flow cytometry cTnT expression in hPSC-CMs for control (CTRL) differentiation samples compared to unpooled reseed samples. Points represent the standard deviation values of 3-4 technical replicates for 2 independent differentiations in GiWi iPSC lines (2 IMR90-4). **F)** Within differentiation standard deviation in flow cytometry cTnT expression in hPSC-CMs for control (CTRL) differentiation samples compared to pooled reseed samples. Points represent the standard deviation values of 3-4 technical replicates for 6 independent differentiations in GiWi lines (4 IMR90-4, 1 H9, 1 19-9-11). P-values for A and D from two-factor linear mixed-effects model with repeated measures testing for a main column effect with a Dunnett's post-hoc test. P-values for B, C, E, and F from two-way ANOVA testing for a main column effect with a Dunnett's post-hoc test. All data are represented as mean ± SD.

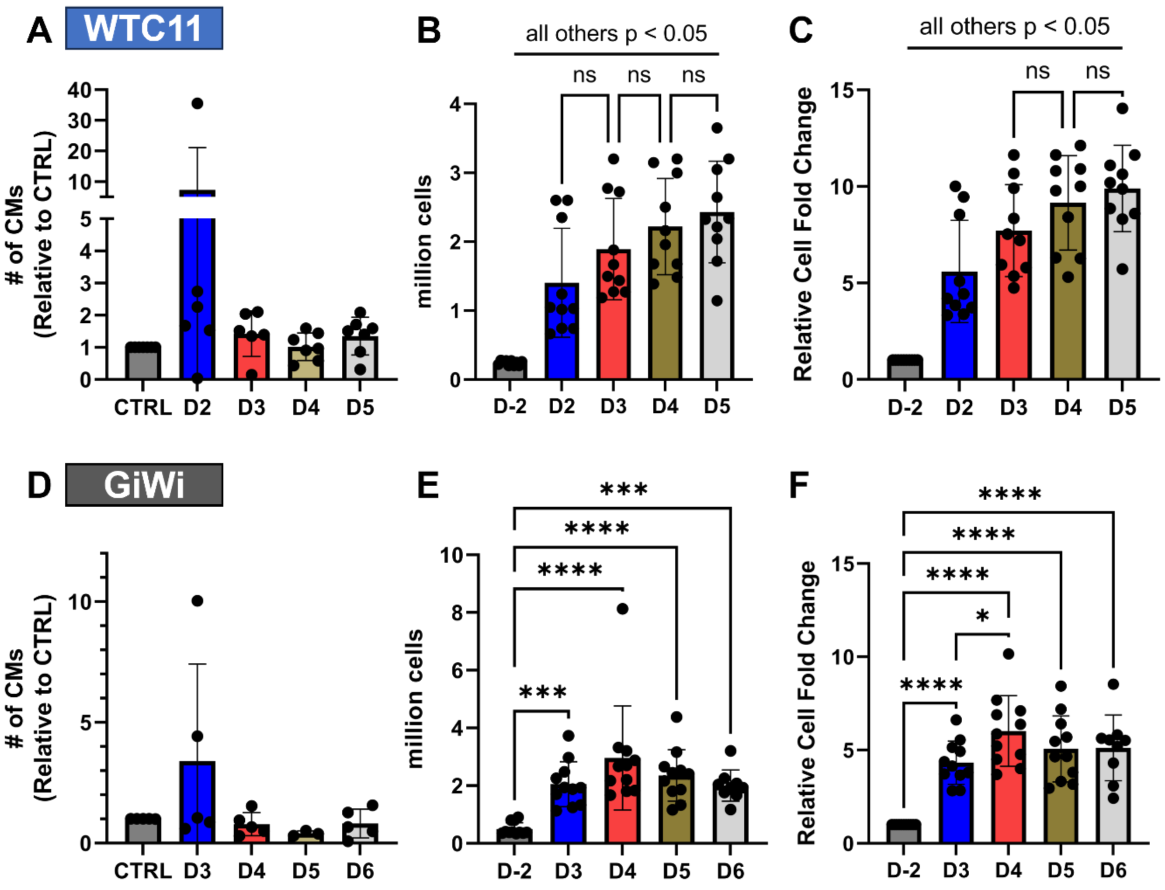

G

Cell Line	Count (million cells)	Day -2	Day 2	Day 3	Day 4	Day 5
WTC11	Count (per lifted 12 well)	0.24 ± 0.03	1.40 ± 0.79	1.89 ± 0.74	2.22 ± 0.70	2.43 ± 0.74
	Reseed Count (1:2.5 SA)	-	0.56	0.76	0.89	0.97
GiWi lines		Day -2	Day 3	Day 4	Day 5	Day 6
	Count (per lifted 12 well)	0.50 ± 0.22	2.05 ± 0.78	2.96 ± 0.18	2.35 ± 0.89	2.00 ± 0.55
	Reseed Count (1:2.5 SA)	-	0.82	1.18	0.94	0.80

Figure S6 (Related to Figure 2). Relative number of CMs by manual counts and live cell counts on the day of reseeding and differentiation initiation. A) The number of CMs relative to CTRL differentiation samples (manual counts). Points represent the mean values of 2-4 technical replicates for 7 independent differentiations in the WTC11 iPSC line. **B)** Visual cell counts (million cells per 12 well) on the indicated days of WTC11 iPSC line CM differentiation. Points represent the mean values of 1-4 technical replicates for 10 independent differentiations. **C)** Relative cell count fold change normalized to D-2 (differentiation seeding date) on the indicated days of WTC11 iPSC line CM differentiation. Points represent the mean values of 1-4 technical replicates for 10 independent differentiations. **D)** The number of CMs relative to CTRL differentiation samples (manual counts). Points represent the mean values of 3-4 technical replicates for 5 independent differentiations in GiWi lines (4 IMR90-4 and 1 19-9-11). **E)** Visual cell counts (million cells per 12 well) on the indicated days of GiWi line CM differentiation. Points represent the mean values of 1-4 technical replicates for 9-11 independent differentiations (8 IMR90-4, 2 H9, and 1 19-9-11). **F)** Relative cell count fold change normalized to D-2 (differentiation seeding date) on the indicated days of GiWi line CM differentiation. Points represent the mean values of 1-4 technical replicates for 9-11 independent differentiations (8 IMR90-4, 2 H9, and 1 19-9-11). **G)** Visual cell counts (million cells per 12 well) ± standard deviation on differentiation seeding (Day -2) and visual cell counts (million cells per 12 well) for each day of progenitor reseeding in WTC11 and GiWi iPSC lines. Reseed cell counts are equal to the lifted 12 well cell count divided by 2.5 to account for 2.5 split ratio. N=10 differentiations for WTC11 and N=11 for GiWi iPSC lines with 1-4 technical replicates. P-values from A and D from two-factor linear mixed-effects model with repeated measures testing for a main column effect with a Dunnett's post-hoc test. All other P-values from analyzing mean values using a two-way ANOVA with Tukey's post-hoc. All data are represented as mean ± SD.

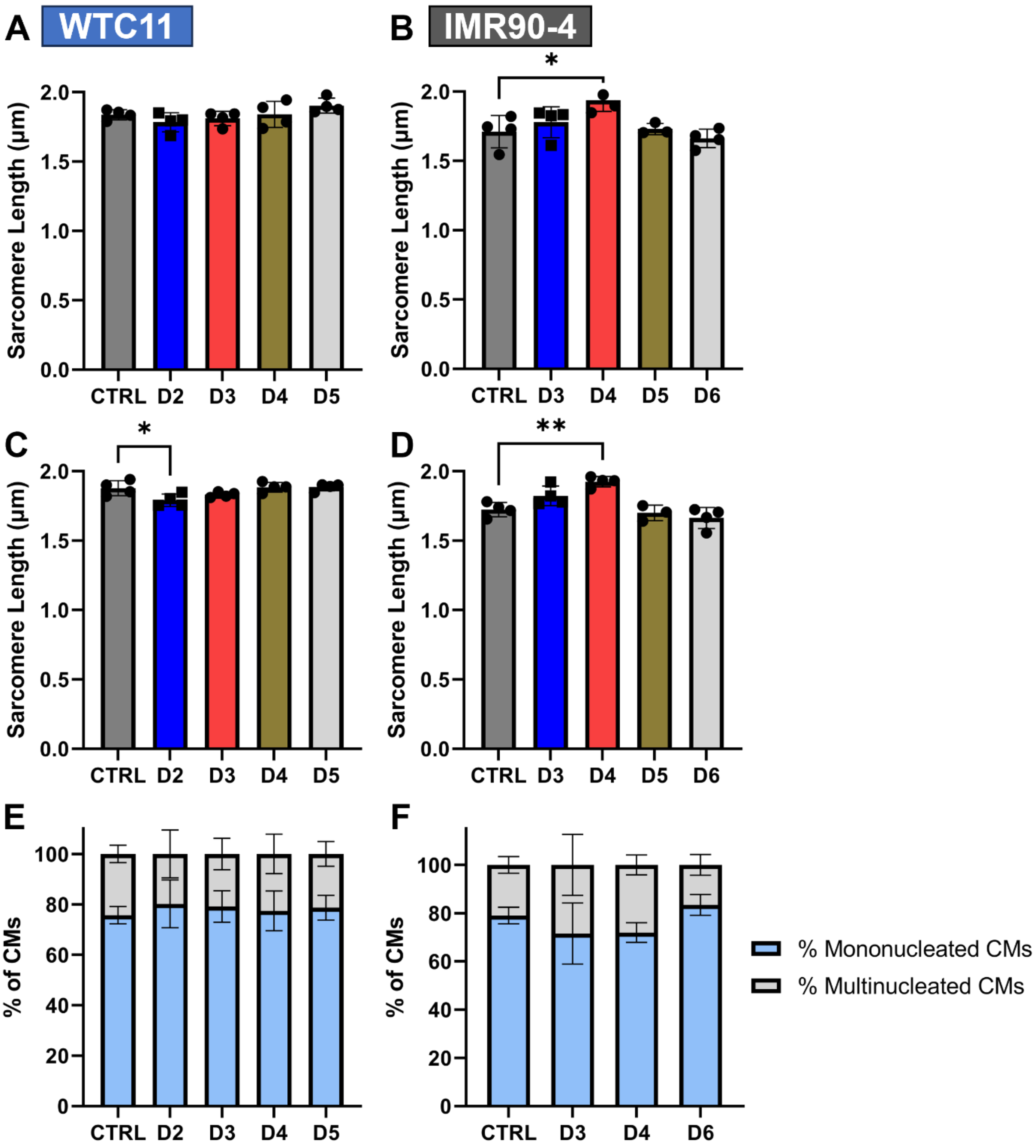

A
WTC11
B
IMR90-4
Sarcomere Length (µm)
2.0
1.5
1.0
0.5
0.0
CTRL
D2
D3
D4
D5
D6
*
C
D
**
E
F
% of CMs
100
80
60
40
20
0
% Mononucleated CMs
% Multinucleated CMs

Figure S7 (Related to Figure 3). Automated sarcomere length quantification and manual multinucleation quantification. A) Sarcomere lengths in the WTC11 iPSC line quantified at 60x magnification using SotaTool. Points represent the mean values of 4 image replicates for one differentiation. **B)** Sarcomere lengths in the IMR90-4 iPSC line quantified at 60x magnification using SotaTool. Points represent the mean values of 3-4 image replicates for one differentiation. **C)** Sarcomere lengths in the WTC11 iPSC line quantified at 40x magnification using SotaTool. Points represent the mean values of 4 well replicates with 4 images per well for one differentiation. **D)** Sarcomere lengths in the IMR90-4 iPSC line quantified at 40x magnification using SotaTool. Points represent the mean values of 4 well replicates with 4 images per well for one differentiation. **E)** Proportions of mononucleated (blue) and multinucleated (gray) CMs in the WTC11 iPSC line quantified at 20x magnification. Points represent the values of 4 image replicates for one differentiation. **F)** Proportions of mononucleated (blue) and multinucleated (gray) CMs in the IMR90-4 iPSC line quantified at 20x magnification. Points represent the values of 4 image replicates for one differentiation. All P-values from one-way ANOVA with Dunnett's post-hoc. All data are represented as mean ± SD.

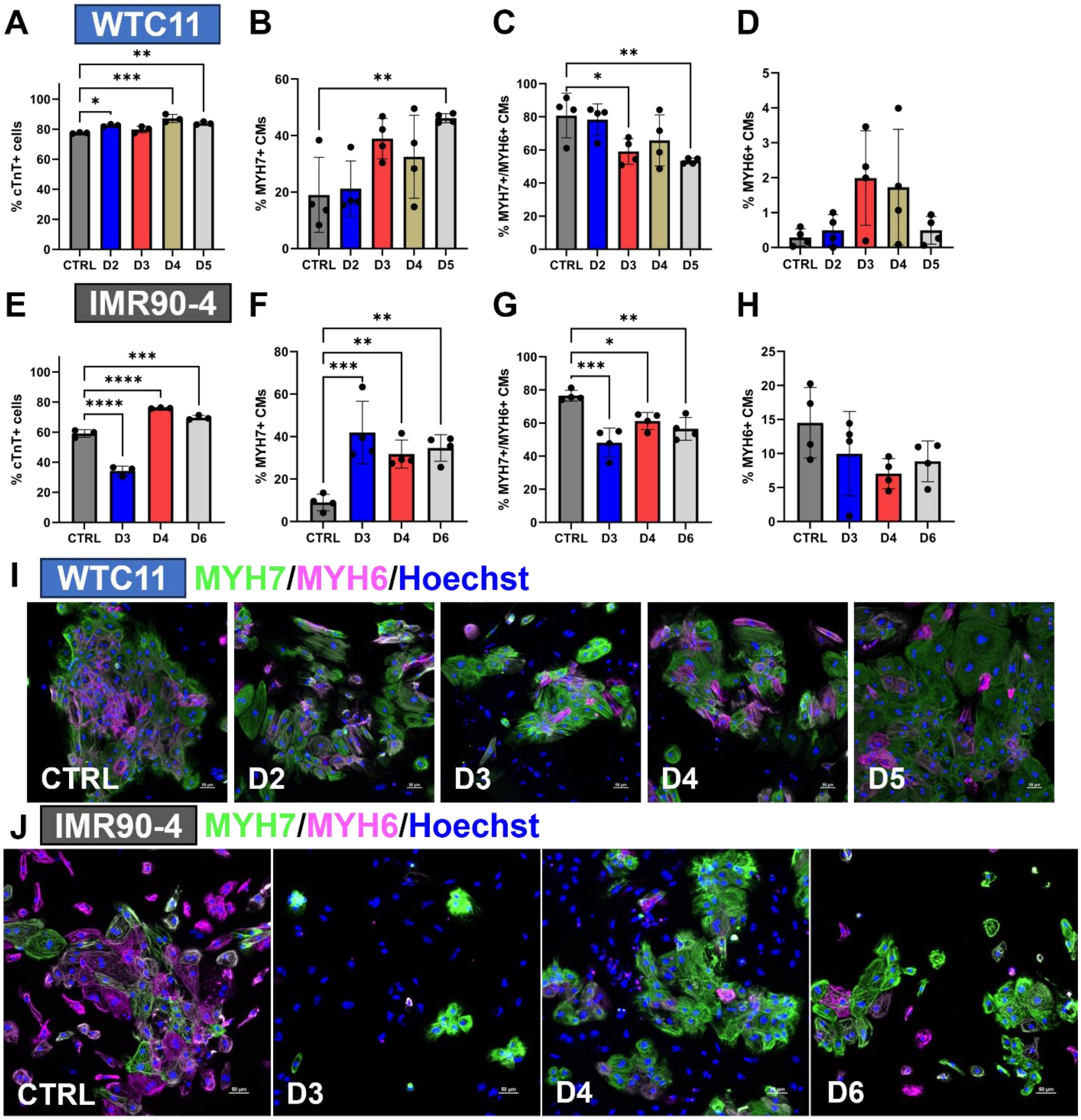

A
WTC11
B
C
D
% cTnT+ cells
% MYH7+ CMs
% MYH7+/MYH6+ CMs
% MYH6+ CMs
CTRL D2 D3 D4 D5
E
IMR90-4
F
G
H
CTRL D3 D4 D6
I
WTC11 MYH7/MYH6/Hoechst
CTRL
D2
D3
D4
D5
J
IMR90-4 MYH7/MYH6/Hoechst
CTRL
D3
D4
D6

Figure S8 (Related to Figure 3). MYH7 and MYH6 immunofluorescence imaging of D23 CMs. A-D) Percentages of **A)** CMs via cTnT flow cytometry, **B)** single positive MYH7+ CMs, **C)** double positive MYH7+/MYH6+ CMs, and **D)** single positive MYH6+ CMs identified by CM nuclear area in the WTC11 iPSC line. Points represent the values of 4 image replicates for one differentiation. **E-H)** Percentages of **E)** CMs via cTnT flow cytometry, **F)** single positive MYH7+ CMs, **G)** double positive MYH7+/MYH6+ CMs, and **H)** single positive MYH6+ CMs identified by CM nuclear area in the IMR90-4 iPSC line. Points represent the mean values of 4 image replicates for one differentiation. **I-J)** Representative immunofluorescence images with MYH6 (magenta), MYH7 (green), Hoechst nuclear (blue) stains in the **G)** WTC11 iPSC line and the **H)** IMR90-4 iPSC line. All images are at 20x magnification with scale bars = 50 μm. All P-values from one-way ANOVA with Dunnett's post-hoc. All data are represented as mean ± SD.

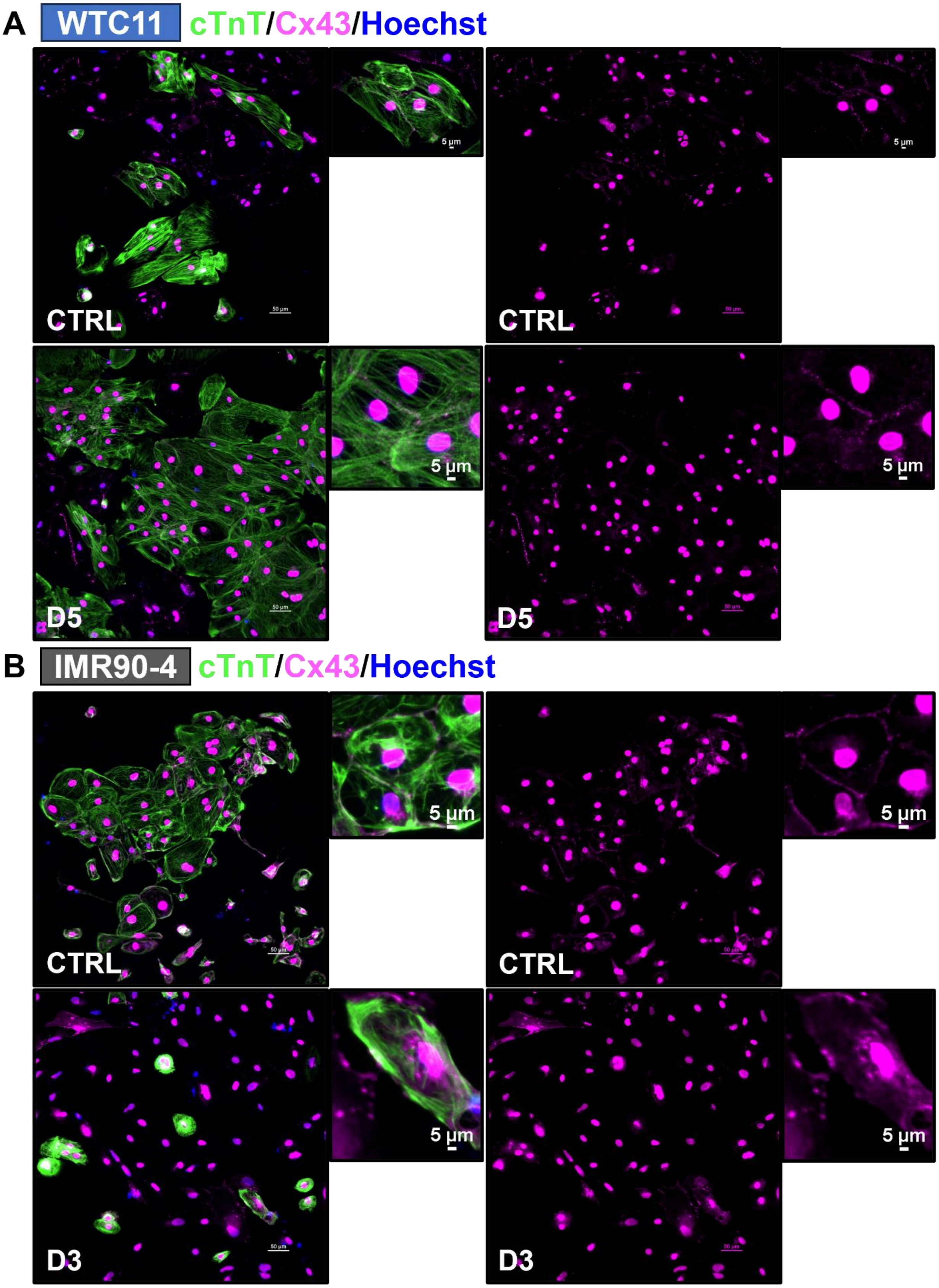
A
WTC11
cTnT/Cx43/Hoechst
CTRL
CTRL
D5
D5
5 µm
5 µm
5 µm
5 µm
B
IMR90-4
cTnT/Cx43/Hoechst
CTRL
CTRL
D3
D3
5 µm
5 µm
5 µm
5 µm

Figure S9 (Related to Figure 3). Cx43 and cTnT immunofluorescence imaging of D23 CMs. Representative immunofluorescence images with Cx43 (magenta), cTnT (green), and Hoechst nuclear (blue) stains**.** All images are at 20x magnification with scale bars = 50 μm for the full image and 5 μm for the image insets. **A)** Images of CTRL and D5 reseed conditions for the WTC11 iPSC line. **B)** Images of CTRL and D3 reseed conditions for the IMR90-4 iPSC line.

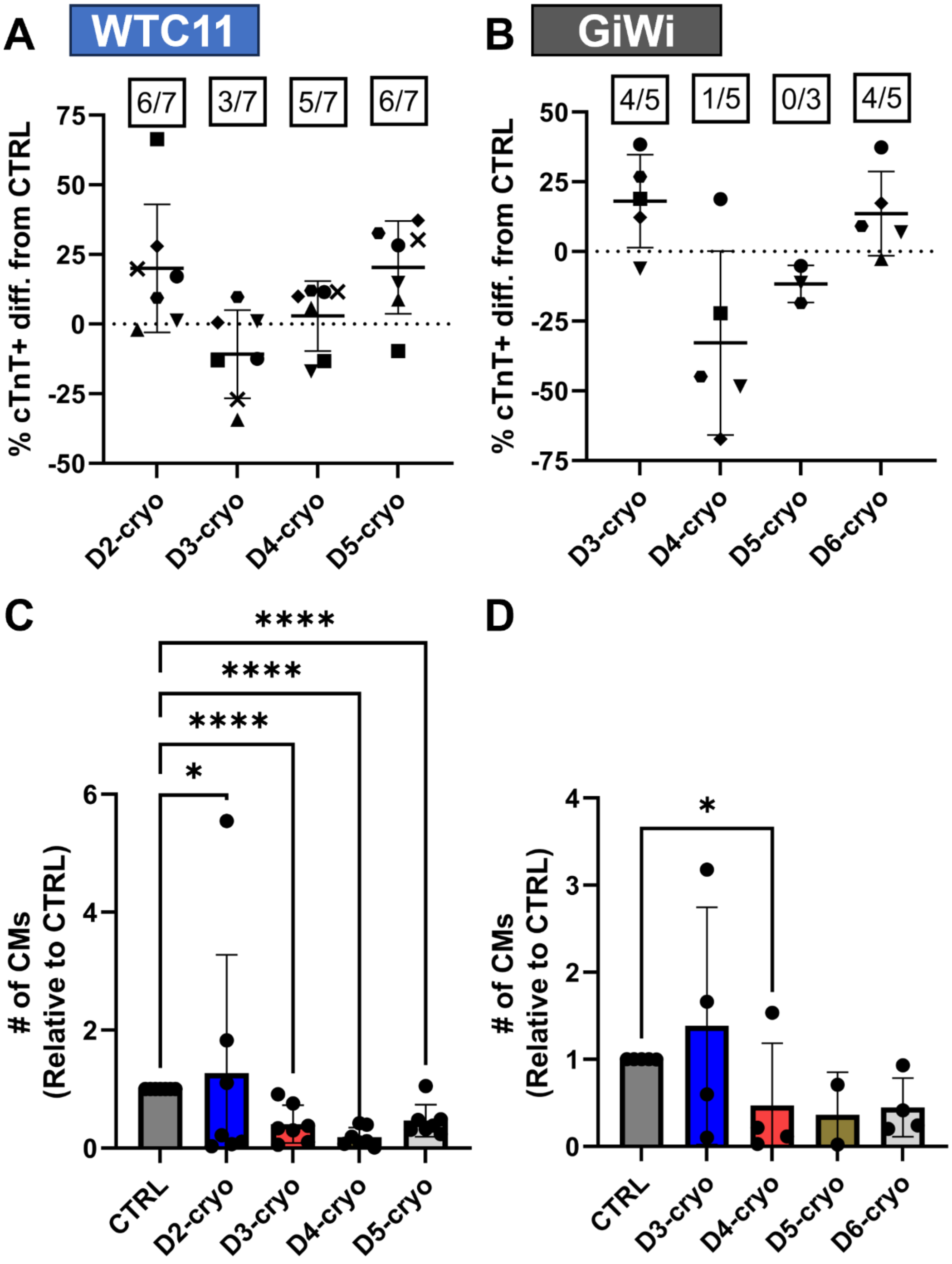

A
WTC11
B
GiWi
6/7
3/7
5/7
6/7
4/5
1/5
0/3
4/5
% cTnT+ diff. from CTRL
D2-cryo
D3-cryo
D4-cryo
D5-cryo
D6-cryo
C
D
****
****
****
*
*
# of CMs (Relative to CTRL)
CTRL

Figure S10 (Related to Figure 4). Difference in cTnT+ purity from CTRL and number of CMs relative to CTRL. A) Absolute difference in the percentage of cTnT+ cells for the indicated day of cryopreserved reseeding compared to the CTRL of the same differentiation. Unique symbols represent 7 independent WTC11 iPSC differentiations. Fractions at the top of the graph represent the number of differentiations out of the total number of differentiations that increased in percentage of cTnT+ cells (difference from CTRL > 0). **B)** Absolute difference in the percentage of cTnT+ cells for the indicated day of cryopreserved reseeding compared to the CTRL of the same differentiation. Unique symbols represent 6 independent GiWi line differentiations (5 IMR90-4, 1 19-9-11). Fractions at the top of the graph represent the number of differentiations out of the total number of differentiations that increased in percentage of cTnT+ cells (difference from CTRL > 0). **C)** The number of CMs relative to CTRL differentiation samples. Points represent the mean values of 3-4 technical replicates for 7 independent differentiations in the WTC11 iPSC line. **D)** The number of CMs relative to CTRL differentiation samples. Points represent the mean values of 2-6 technical replicates for 5 independent GiWi line differentiations (4 IMR90-4, 1 19-9-11). All P-values from two-factor linear mixed-effects model with repeated measures with a Dunnett's post-hoc test (full model effect for A and main column effect for D). All data are represented as mean ± SD.

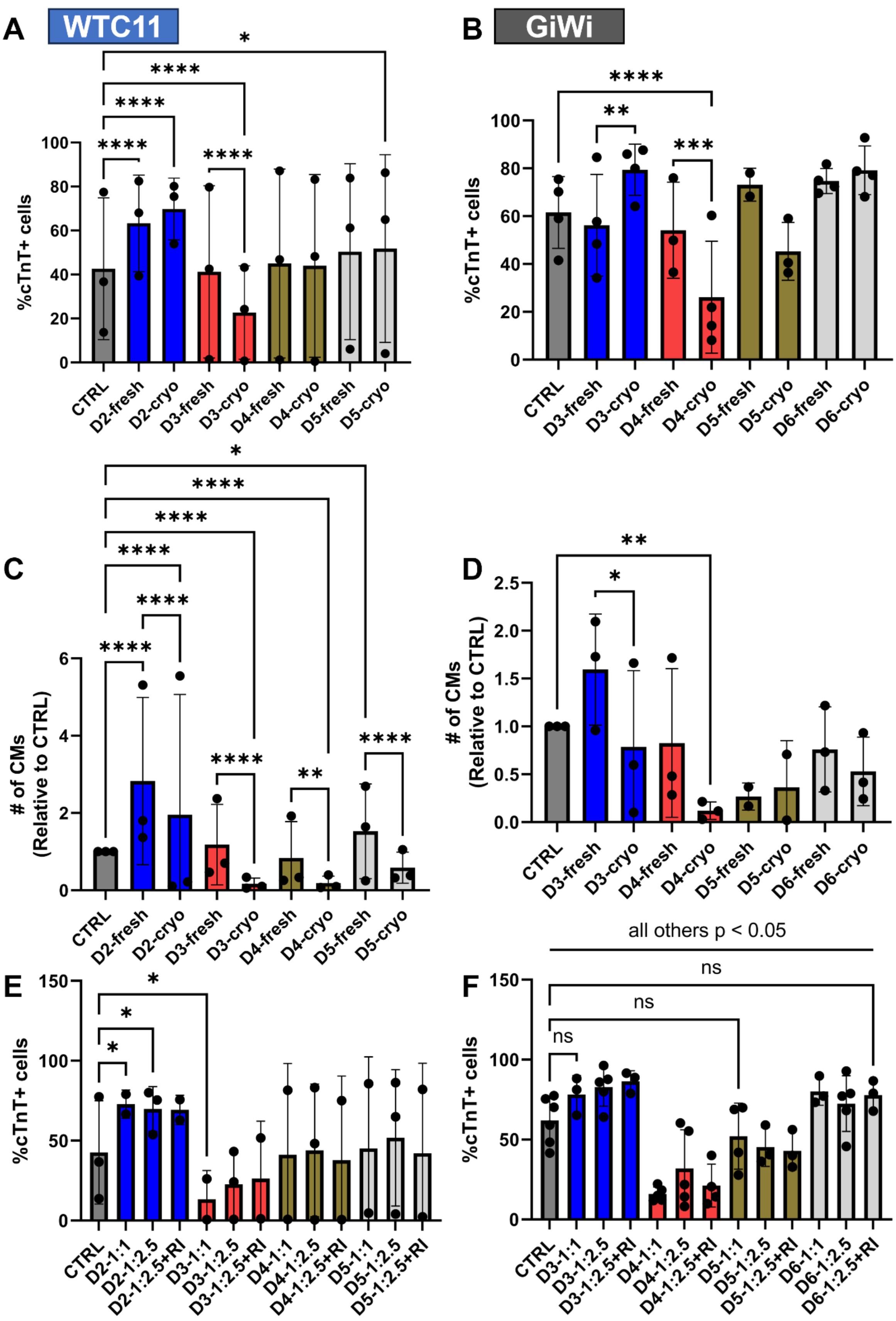
A
WTC11
B
GiWi
C
D
E
F
%cTnT+ cells
# of CMs (Relative to CTRL)
CTRL
D2-fresh
D2-cryo
D3-fresh
D3-cryo
D4-fresh
D4-cryo
D5-fresh
D5-cryo
D6-fresh
D6-cryo
D2-1:1
D2-1:2.5
D2-1:2.5+RI
D3-1:1
D3-1:2.5
D3-1:2.5+RI
D4-1:1
D4-1:2.5
D4-1:2.5+RI
D5-1:1
D5-1:2.5
D5-1:2.5+RI
D6-1:1
D6-1:2.5
D6-1:2.5+RI
all others p < 0.05
ns

Figure S11 (Related to Figure 4). CM purity and CM number for fresh versus cryopreserved reseeding and additional cryopreservation media conditions. A) Flow cytometry analysis of cTnT expression in hPSC-CMs for control (CTRL) differentiation samples compared to fresh and cryopreserved reseed samples (1:2.5 by surface area) at the indicated days in the WTC11 iPSC line. Points represent the mean values of 2-4 technical replicates for 3 independent differentiations. **B)** Flow cytometry analysis of cTnT expression in hPSC-CMs for control (CTRL) differentiation samples compared to fresh and cryopreserved reseed samples (1:2.5 by surface area) at the indicated days in GiWi lines. Points represent the mean values of 3-6 technical replicates for 4 independent differentiations (3 IMR90-4, 1 19-9-11). **C)** The number of CMs relative to CTRL differentiation samples. Points represent the mean values of 2-4 technical replicates for 3 independent differentiations in the WTC11 iPSC line. **D)** The number of CMs relative to CTRL differentiation samples. Points represent the mean values of 3-4 technical replicates for 3 independent differentiations in GiWi lines (2 IMR90-4, 1 19-9-11). **E)** Flow cytometry analysis of cTnT expression in hPSC-CMs for control (CTRL) differentiation samples compared to cryopreserved samples at the indicated days and surface area split ratios with or without Y-27632 at 5 μM (RI) in the WTC11 iPSC line. Points represent the mean values of 2-4 technical replicates for 3 independent differentiations. **F)** Flow cytometry analysis of cTnT expression in hPSC-CMs for control (CTRL) differentiation samples compared to cryopreserved samples at the indicated days and surface area split ratios with or without Y-27632 at 5 μM (RI) in GiWi lines. Points represent the mean values of 2-6 technical replicates for 6 independent differentiations (5 IMR90-4, 1 19-9-11). P-values for A, C from two-factor linear mixed-effects model with repeated measures testing for a full model effect with a Tukey's post-hoc test. P-values for B, D from two-factor linear mixed-effects model with repeated measures testing for a main column effect with a Tukey's post-hoc test. P-values for E-F from two-factor linear mixed-effects model with repeated measures testing for a main column effect with a Dunnett's post-hoc test. All data are represented as mean ± SD.

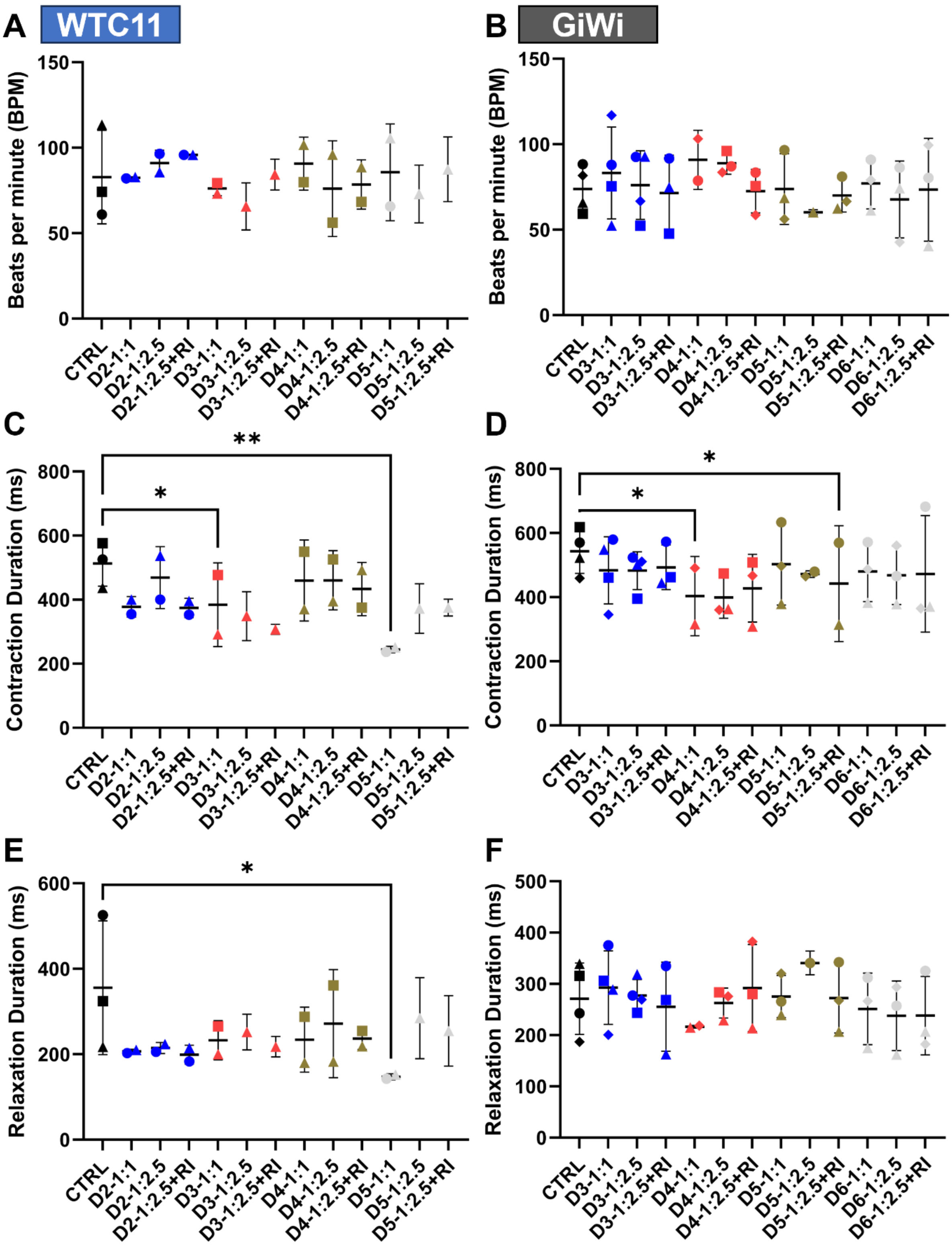
A
WTC11
B
GiWi
Beats per minute (BPM)
150
100
50
0
CTRL
D2-1:1
D2-1:2.5
D2-1:2.5+RI
D3-1:1
D3-1:2.5
D3-1:2.5+RI
D4-1:1
D4-1:2.5
D4-1:2.5+RI
D5-1:1
D5-1:2.5
D5-1:2.5+RI
D6-1:1
D6-1:2.5
D6-1:2.5+RI
C
D
Contraction Duration (ms)
800
600
400
200
**
*
E
F
Relaxation Duration (ms)
500
300

Figure S12 (Related to Figure 4). Contractile parameters for altered cryopreserved media and reseeding ratio conditions. A, C, E) Contractile parameters (beats per minute – **A**, contraction duration - **C**, and relaxation duration - **E**) in the WTC11 iPSC line quantified using MUSCLEMOTION. Points represent the mean values of 1-5 technical replicates for 3 independent differentiations, which are represented by unique symbols. **B, D, F)** Contractile parameters (beats per minute – **B**, contraction duration - **D**, and relaxation duration - **F**) in GiWi lines quantified using MUSCLEMOTION. Points represent the mean values of 1-5 technical replicates for 4 independent differentiations, which are represented by unique symbols (3 IMR90-4, 1 19-9-11). All P-values from two-factor linear mixed-effects model with repeated measures testing for a main column effect with a Dunnett's post-hoc test. All data are represented as mean ± SD.

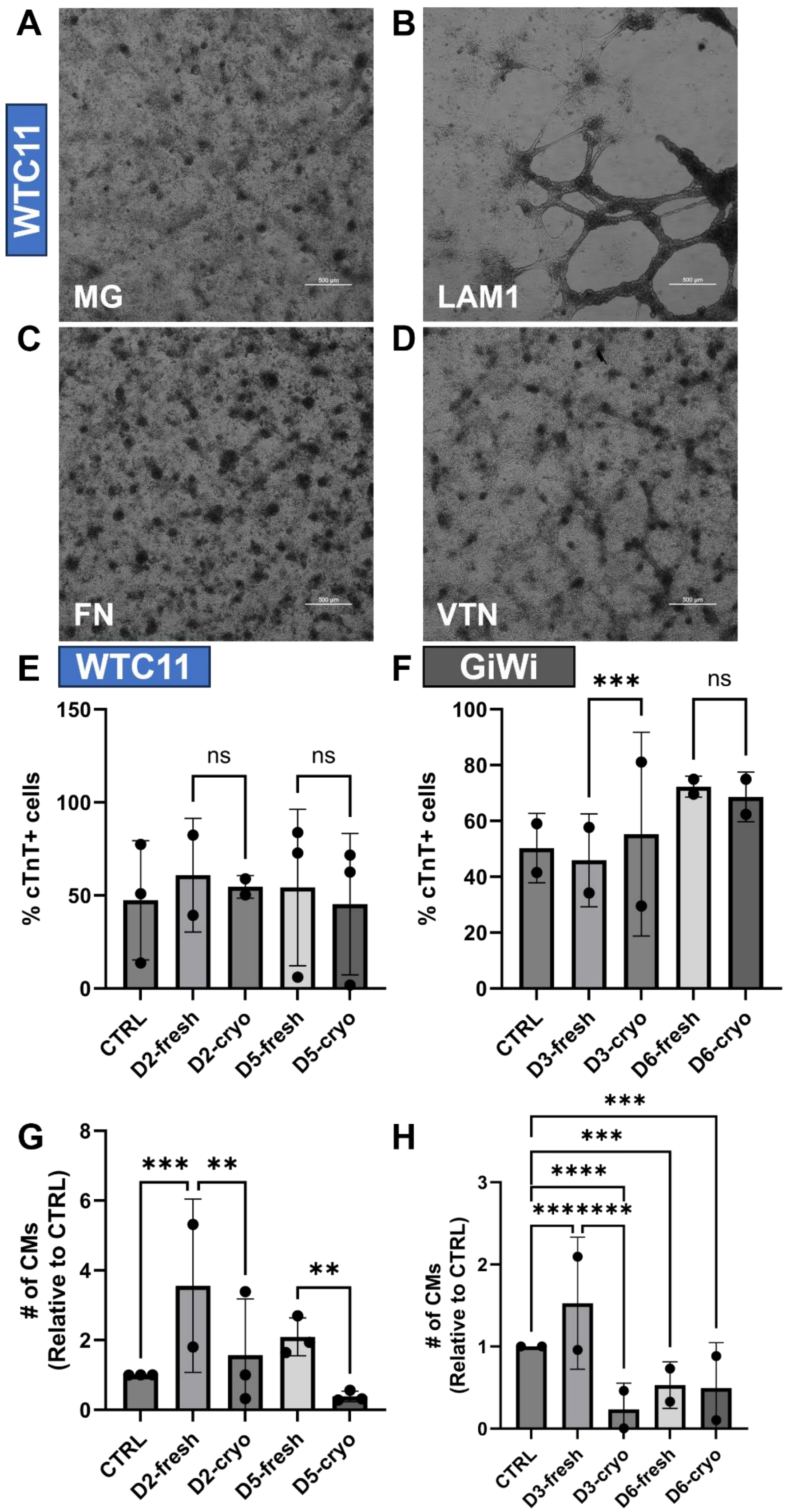
A
B
C
D
WTC11
MG
LAM1
FN
VTN
E
WTC11
F
GiWi
% cTnT+ cells
ns
ns
***
ns
CTRL
D2-fresh
D2-cryo
D5-fresh
D5-cryo
D3-fresh
D3-cryo
D6-fresh
D6-cryo
G
H
# of CMs
(Relative to CTRL)
***
**
****
****

Figure S13 (Related to Figure 5). Cell recovery after cryopreserved plating of mesoderm progenitors and CPCs on different ECMs and CM purity and number for fresh versus cryopreserved conditions. A) Representative phase contrast image of D16 CMs in the WTC11 iPSC line following cryopreserved reseeding onto Matrigel on D5 (D16 cTnT purity for CTRL (not shown) = 76.5% and for CPC Matrigel Reseed = 83.6%). **B)** Representative phase contrast image of D16 CMs in the WTC11 iPSC line following cryopreserved reseeding onto Laminin-111 on D5 (D16 cTnT purity = 93.0%). **C)** Representative phase contrast image of D16 CMs in the WTC11 iPSC line following cryopreserved reseeding onto Fibronectin on D5 (D16 cTnT purity = 67.6%). **D)** Representative phase contrast image of D16 CMs in the WTC11 iPSC line following cryopreserved reseeding onto VTN on D5 (D16 cTnT purity = 77.7%). All images are at 4x magnification with scale bars = 500 μm. **E)** Flow cytometry analysis of cTnT expression in hPSC-CMs for control (CTRL) differentiation samples compared to fresh and cryopreserved reseed samples (1:2.5 by surface area) at the indicated days in the WTC11 iPSC line. Points represent the mean values of 3 technical replicates for 3 independent differentiations. **F)** Flow cytometry analysis of cTnT expression in hPSC-CMs for control (CTRL) differentiation samples compared to fresh and cryopreserved reseed samples (1:2.5 by surface area) at the indicated days in GiWi lines. Points represent the mean values of 3 technical replicates for 2 independent differentiations (1 IMR90-4, 1 19-9-11). **G)** The number of CMs relative to CTRL differentiation samples. Points represent the mean values of 3 technical replicates for 3 independent differentiations in the WTC11 iPSC line. **H)** The number of CMs relative to CTRL differentiation samples. Points represent the mean values of 3 technical replicates for 2 independent differentiations in GiWi lines (1 IMR90-4, 1 19-9-11). All P-values from two-factor linear mixed-effects model with repeated measures testing for a main column effect with a Tukey's post-hoc test. All data are represented as mean ± SD.

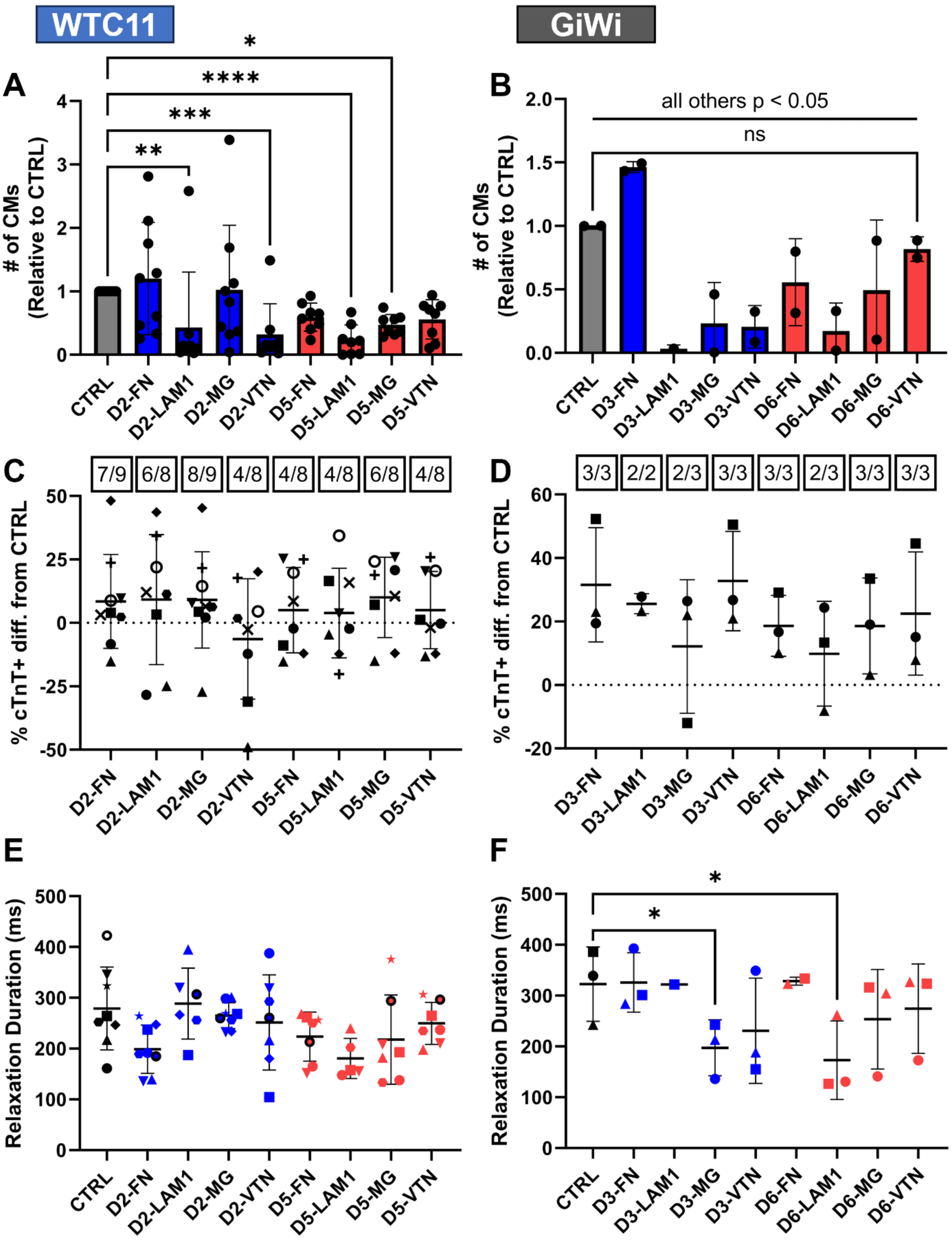
WTC11
GiWi
A
*
****
***
**
# of CMs (Relative to CTRL)
CTRL
D2-FN
D2-LAM1
D2-MG
D2-VTN
D5-FN
D5-LAM1
D5-MG
D5-VTN
B
all others p < 0.05
ns
D3-FN
D3-LAM1
D3-MG
D3-VTN
D6-FN
D6-LAM1
D6-MG
D6-VTN
C
7/9
6/8
8/9
4/8
4/8
4/8
6/8
4/8
% cTnT+ diff. from CTRL
D
3/3
2/2
2/3
3/3
3/3
2/3
3/3
3/3
E
Relaxation Duration (ms)
F
*
*

Figure S14 (Related to Figure 5). CM purity, CM number, and contractile properties for cryopreserved reseeding on different ECMs. A) The number of CMs relative to CTRL differentiation samples. Points represent the mean values of 2-4 technical replicates for 9 independent differentiations in the WTC11 iPSC line. **B)** The number of CMs relative to CTRL differentiation samples. Points represent the mean values of 3 technical replicates for 2 independent differentiations in GiWi lines (1 IMR90-4, 1 19-9-11). **C)** Absolute difference in the percentage of cTnT+ cells for the indicated ECM and day of cryopreserved reseeding compared to the CTRL of the same differentiation. Unique symbols represent 9 independent WTC11 iPSC differentiations. Fractions at the top of the graph represent the number of differentiations out of the total number of differentiations that increased in percentage of cTnT+ cells (difference from CTRL > 0). **D)** Absolute difference in the percentage of cTnT+ cells for the indicated ECM and day of cryopreserved reseeding compared to the CTRL of the same differentiation. Unique symbols represent 3 independent GiWi line differentiations (2 IMR90-4, 1 19-9-11). Fractions at the top of the graph represent the number of differentiations out of the total number of differentiations that increased in percentage of cTnT+ cells (difference from CTRL > 0). **E)** Relaxation duration in the WTC11 iPSC line quantified using MUSCLEMOTION. Points represent the mean values of 1-4 technical replicates for 8independent differentiations, which are represented by unique symbols. **F)** Relaxation duration in the GiWi lines quantified using MUSCLEMOTION. Points represent the mean values of 1-4 technical replicates for 3 independent differentiations, which are represented by unique symbols (2 IMR90-4, 1 19-9-11). All P-values from Two-factor linear mixed-effects model with repeated measures testing for a main column effect with a Dunnett's post-hoc test. All data are represented as mean ± SD.
